# Genetic architecture of tuber-bound free amino acids in potato and effect of growing environment on the amino acid content

**DOI:** 10.1038/s41598-023-40880-5

**Published:** 2023-08-25

**Authors:** Jeewan Pandey, Dalton Thompson, Madhumita Joshi, Douglas C. Scheuring, Jeffrey W. Koym, Vijay Joshi, M. Isabel Vales

**Affiliations:** 1 Department of Horticultural Sciences, Texas A&M University, College Station, TX 77843 USA; 2 Texas A&M AgriLife Research and Extension Center, Uvalde, TX 78801 USA; 3 Texas A&M AgriLife Research and Extension Center, Lubbock, TX 79403 USA

**Keywords:** Plant breeding, Genome-wide association studies

## Abstract

Free amino acids in potato tubers contribute to their nutritional value and processing quality. Exploring the natural variation in their accumulation in tubers across diverse genetic backgrounds is critical to potato breeding programs aiming to enhance or partition their distribution effectively. This study assessed variation in the tuber-bound free amino acids in a diversity panel of tetraploid potato clones developed and maintained by the Texas A&M Potato Breeding Program to explore their genetic basis and to obtain genomic-estimated breeding values for applied breeding purposes. Free amino acids content was evaluated in tubers of 217 tetraploid potato clones collected from Dalhart, Texas in 2019 and 2020, and Springlake, Texas in 2020. Most tuber amino acids were not affected by growing location, except histidine and proline, which were significantly lower (− 59.0%) and higher (+ 129.0%), respectively, at Springlake, Texas (a location that regularly suffers from abiotic stresses, mainly high-temperature stress). Single nucleotide polymorphism markers were used for genome-wide association studies and genomic selection of clones based on amino acid content. Most amino acids showed significant variations among potato clones and moderate to high heritabilities. Principal component analysis separated fresh from processing potato market classes based on amino acids distribution patterns. Genome-wide association studies discovered 33 QTL associated with 13 free amino acids. Genomic-estimated breeding values were calculated and are recommended for practical potato breeding applications to select parents and advance clones with the desired free amino acid content.

## Introduction

Potato is a major dietary staple crop consumed worldwide. Potato tubers are a rich source of carbohydrates (mainly starch), dietary fiber, high-quality protein, vitamins, antioxidants, and minerals^1,2^. The total protein content of potato tubers is relatively low (1.0–3.0% FW or 5.0–14.0% DW) but comparable to cereals on a dry basis and better than commonly consumed vegetables^3^. Potato protein has high biological activity due to its high free amino acid score (69.0–76.0%)^4,5^ and higher nutritional value than other commonly consumed plant sources due to its high content of essential amino acids^6^. Potato contains all nine essential amino acids^3^, qualitatively superior to many other plant-based proteins^7^. Nearly half of the total amino acids in potatoes are in the free form^8,9^. Asparagine and glutamine amides dominate the free amino acids (34.0–90.0%)^10,11^, whereas lysine, tyrosine, and the sulfur-containing amino acids methionine and cysteine are still limited in potatoes^3,12^. Nevertheless, potato tubers contain higher amounts of lysine than cereals^13^. Enhancing the protein quality of potato tubers by traditional breeding or genetic manipulation of pathways involved in the metabolism of limiting amino acids holds the potential to improve potato’s biological activity and nutritional value and thus enhance human health and nutrition.

Free amino acids react with reducing sugars during frying, resulting in a non-enzymatic browning reaction (Maillard) which affects the organoleptic and other quality traits of processed potato products^14,15^. Asparagine reacts with reducing sugars to form a carcinogenic compound called acrylamide^16,17^, a major concern in processed potato products^18–22^. Genetically modified (GM) potatoes produced by manipulating the expression of the *asparagine synthetase* gene using RNA interference have low asparagine content and reduced acrylamide levels after processing^23,24^. Free asparagine and reducing sugars are precursors for acrylamide formation via deamination and decarboxylation processes^16,17,25^. In potatoes, the precursor concentration plays a significant role in determining the acrylamide-forming potential^18,20–22,26–28^. A study reported a significant correlation between free asparagine concentration and acrylamide-forming potential in French fry varieties^18,28^. Despite the need, polyploidy and high heterozygosity make breeding and selection of low acrylamide-forming potential potato cultivars for healthy fries and chips challenging. Possibilities of using the Clustered Regularly Interspaced Short Palindromic Repeat (CRISPR) system to manipulate reducing sugars and aspargine metabolism in potatoes are already being considered^29^. Another amino acid affecting flavor is methionine, a precursor of methional, a major volatile associated with the aroma of potato chips and french fries^30^. Enhancing free methionine in tubers for nutritional and aroma benefits is of interest to the food industry and consumers. Transgenic approaches to improve free methionine levels in potato tubers have been used but had, in several cases, undesired effects, including morphological changes in tubers or plants^31–37^. Given the uncertainty about the acceptance of GM potatoes, selecting low asparagine and high methionine in potato tubers through conventional breeding could be the way to develop healthy and flavorful potato varieties. Most studies addressing quality traits focus on increasing starch content^38–40^, diminishing reducing sugars^41,42^, and possessing glycoalkaloid levels below toxic limits^43,44^ in potato tubers. Increasing the protein content in potato tubers is also desirable. However, breeding for more free amino acids in potato tubers may favor indirect selection for low carbohydrate levels because of their inverse relationship with protein content^45^.

Significant genetic variation in the relative amounts of free proteinogenic and non-proteinogenic amino acids has been observed in cultivated potatoes and landraces^46–48^. Screening of a diploid potato population using targeted^49^ and untargeted^46^ metabolite analysis showed significant variation in amino acid content, explaining the underlying genetic variation and identifying metabolic quantitative trait loci (QTL). Due to technological advancements in genotyping, genome-wide association studies (GWAS) have evolved as a powerful tool for exploring the genetic basis of various traits in several crop species, including potatoes^50–58^. A GWAS study performed using the metabolome of cooked potatoes of the Solanaceae Coordinated Agricultural Project (SolCAP) diversity panel^59^ identified several metabolites, including five free amino acids, significantly associated with SNP markers^60^. Evaluating phenotypic variation for free amino acid levels in diversity panels used by breeding programs should provide a baseline to plan selection strategies to improve potatoes’ nutritional properties. Diversity panels can be used to understand the genetic basis of the traits using GWAS and generate genomic-estimated breeding values (GEBVs) of traits using genomic selection (GS) for practical marker-assisted selection in breeding programs. Losses in nitrogen and amino acids in potato tubers differ significantly according to cooking techniques; chips and canned potatoes show the maximum loss, followed by drum-dried and french-fried potatoes^61–64^. Field production practices, growing environmental conditions, nitrogen-fertilization rate and application time, residual soil nitrogen, soil-moisture availability, and storage conditions also influence the amino acid content^65–68^. The broader goal of this research would be to assist the breeding program in developing healthier and more nutritious potatoes using conventional breeding and guide the development of strategies for metabolic engineering. The specific objectives were to i) evaluate the phenotypic variation for free amino acids of raw potato tubers of a diversity panel of tetraploid clones and to assess the effect of field location and year ii) understand the genetic basis of free amino acids using GWAS, and iii) generate GEBVs via GS to facilitate the selection of parents and advancement of clones through the breeding pipeline.

## Results

### Phenotypic variation for free amino acids in potato

**Figure 1:**
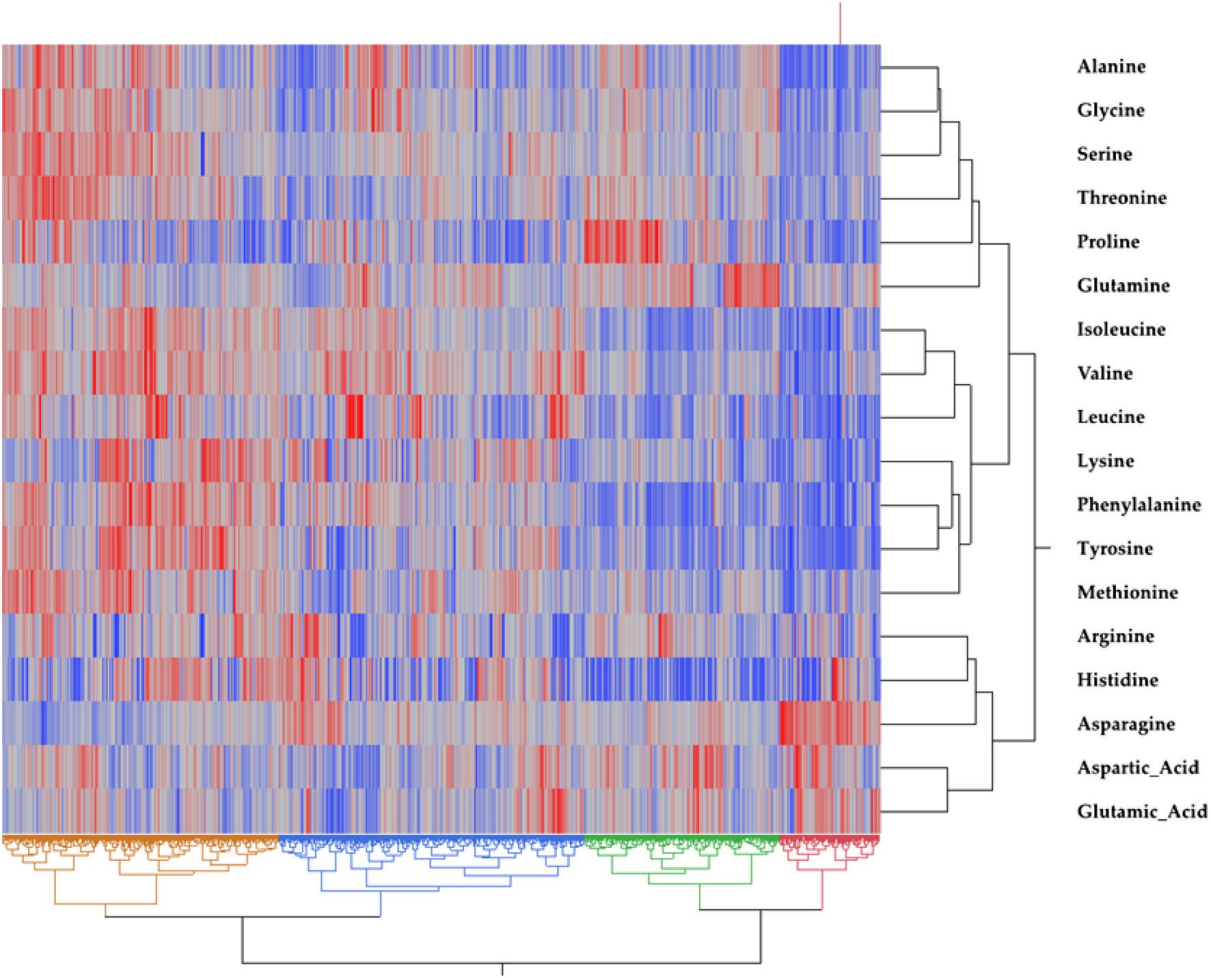
Hierarchical cluster TwoWay Analysis (Ward Method) of free amino acids across 217 tetraploid potato clones. Each clone is visualized as a single column. Each row represents an individual amino acid. Red denotes high amino acid content, whereas blue denotes a low free amino acid content.

The diversity panel (217) of cultivated potato clones evaluated in Dalhart, Texas in 2019 and 2020, and Springlake, Texas in 2020 showed phenotypic variation for the relative amounts of all 19 free amino acids in potato tubers. The phenotypic distributions of amino acid content were normal or close to normal in most cases (Supplementary Fig. 1). Histidine, arginine, asparagine, aspartic acid, alanine, proline, valine, leucine, and phenylalanine distributions differed significantly from normal per Shapiro–Wilk test^69^ (*p* < 0.05) (Supplementary Fig. 1). Hierarchical cluster analysis of free amino acids reflected the metabolic relationship between amino acids (Fig. 1).

**Figure 2:**
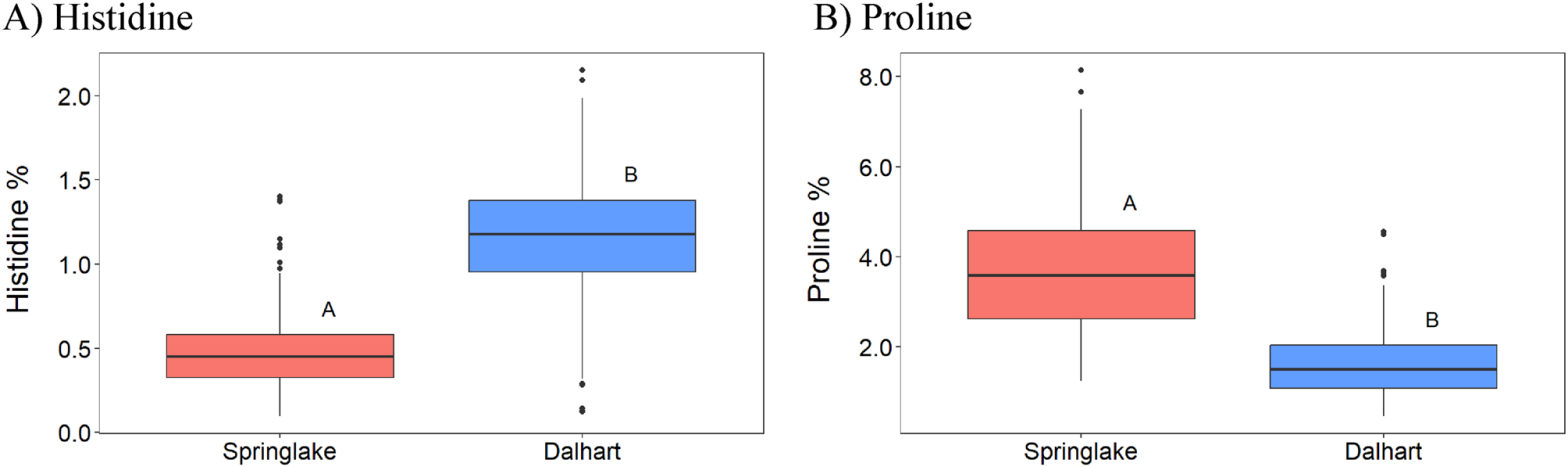
Box-plots for histidine (**A**) and proline (**B**) content in raw potato tubers diversity panel from the two locations (Springlake and Dalhart, TX). The amino acid means of tubers from the diversity panel (217 clones) at each location are represented by bold horizontal lines through the box-plot. Different letters above the boxplots denote significant differences between the means of the two locations based on Student t-test (*p* < 0.05).

For all traits evaluated, the analysis of variance indicated significant differences between clones (Supplementary Table 1). The interactions between clones and environments were significant for most amino acids, except arginine, asparagine, glutamine, threonine, glycine, and lysine. Analysis of variance from the two Texas locations (Dalhart and Springlake) in 2020 indicated that most tuber amino acids were not affected by growing location, except histidine and proline (Fig. 2 and Supplementary Table 2), which were significantly lower (− 59.0%) and higher (+ 129.0%), respectively, at Springlake, TX (a location that regularly suffers from abiotic stresses, mainly high-temperature stress). The average histidine content in tubers at Springlake was 0.5%, whereas tubers harvested from the Dalhart location had an average of 1.2%. In relation to proline, the average in Springlake was 1.6%, whereas the value was 3.7% in Dalhart (Fig. 2). In Springlake, the lowest amount of histidine was observed in Russet Norkotah 112 (0.1%), and the highest was found in ATTX98453-3R (1.4%). In Dalhart, the lowest amount of histidine was observed in ATX08181-5Y/Y (0.1%) the highest was found in ATX9332-8Ru (2.2%) (Supplementary Table 2). Regarding proline, in Springlake, the lowest value was obtained in TX05249-11W (1.3%), and the highest value was obtained in clone NDTX7590-3R (8.1%) (Supplementary Table 2). In Dalhart, the lowest value of proline was found in ATX9332-8Ru (0.5%), and the highest value was observed in AORTX09037-1W/Y (4.6%) (Supplementary Table 2). Analysis of variance with data from 2 years (2019, 2020, Dalhart location) revealed no significant differences between years, but the interaction between clone and year was significant for histidine, serine, and proline (Supplementary Table 1).

Across the diversity panel, asparagine and glutamine were the most abundant amino acids (33.0%, and 21.0%, respectively). In contrast, histidine and glycine were the least abundant, with average relative compositions of (0.8%, and 0.9%, respectively) (Table 1). Asparagine was highest in the clone NDTX060700C-1W (55.0%) and lowest in the clone COTX94216-1R (21.0%) (Supplementary Table 3). The clone ATTX01178-1R had the highest concentration of glutamine (32.0%), whereas COTX08365F-3P/P had the highest concentration of valine (10.7%) (Supplementary Table 3). The highest proportion of methionine (2.5%) was found in the clone ATX9130-1Ru, whereas COTX03134-1Y/W had the highest concentration of lysine (5.6%) (Supplementary Table 3).

**Table 1:** Mean, range, and broad-sense heritability (H^2^) for free amino acids in 217 potato clones evaluated in Dalhart, Texas in 2019 and 2020, and Springlake, Texas in 2020.

Free amino acids^†^ (%)	Mean	Range		H^2^
		Minimum	Maximum	
*Histidine*	0.80	0.02	1.91	0.15
Arginine	3.66	1.25	7.67	0.75
Asparagine	33.33	21.32	55.56	0.79
Glutamine	21.52	11.63	32.00	0.68
Serine	2.68	1.20	4.84	0.86
Glutamic acid	5.76	1.88	10.03	0.64
Aspartic acid	4.54	2.46	7.69	0.58
*Threonine*	2.12	0.63	3.56	0.81
Glycine	0.93	0.28	1.69	0.82
Alanine	2.92	0.77	7.51	0.89
Proline	2.66	1.15	5.28	0.61
*Lysine*	3.25	1.10	5.56	0.62
*Tyrosine*	1.20	0.26	2.19	0.62
*Methionine*	1.46	0.77	2.49	0.77
*Valine*	7.59	3.06	10.68	0.82
*Isoleucine*	2.50	1.15	3.82	0.69
*Leucine*	1.26	0.47	2.42	0.60
*Phenylalanine*	1.54	0.38	3.27	0.74

^†^Free amino acids in italics are essential amino acids.

Significant variation was observed between potato market groups for various free amino acids (Supplementary Table 4). Histidine was significantly higher in purples than in chippings and russets potatoes. The reds had a significantly lower asparagine (30.0%) than chippers (38.9%) and yellows (35.0%). Glutamine was significantly higher in chippers and phenylalanine in reds than in other market groups. Methionine was significantly higher in russets (1.6%) than in chippers (1.4%), yellows (1.4%), and purples (1.3%) (Supplementary Table 4). No significant differences were found between market groups for aspartic acid and lysine contents.

**Figure 3:**
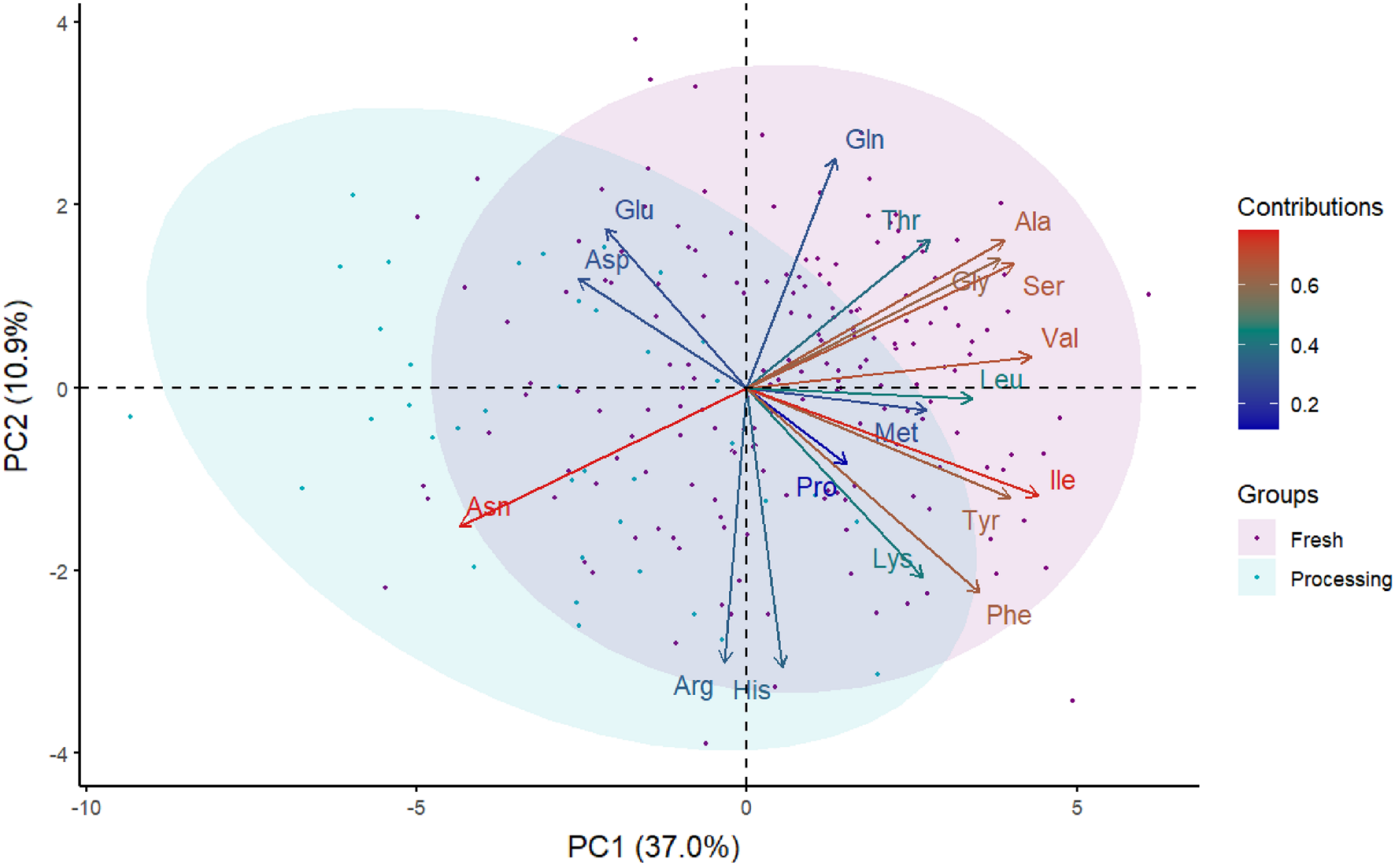
Principal component analysis separating fresh (specific gravity < 1.069) and processing (specific gravity > 1.070) potato market classes based on the free amino acid (His = histidine, Arg = arginine, Asn = asparagine, Gln = glutamine, Ser = serine, Glu = glutamic acid, Asp = aspartic acid, Thr = threonine, Gly = glycine, Ala = alanine, Pro = proline, Lys = lysine, Tyr = tyrosine, Met = methionine, Val = valine, Ile = isoleucine, Leu = leucine, Phe = phenylalanine) composition of 217 potato clones. Each point represents the projection of an individual potato clone in the PC1 and PC2 axes, and the dot colors represent the market group (purple = fresh and cyan = processing). The ellipses represent 95% confidence intervals around the centroid of each data cluster. The contributions (%) of free amino acids to PC1 and PC2 from low to high are indicated by blue to red color gradient, respectively.

The diversity panel of clones was subjected to principal component analysis (PCA) based on free amino acid percentages and separation between fresh and processed (mainly chippers, since most of the russets included in the diversity panel belong to the fresh or dual-purpose) potato market classes. The first two principal components accounted for 47.9% of the total variance in the data, of which first principal component (PC1) explained 37.0% and second principal component (PC2) explained 10.9% of this variance (Fig. 3). Asparagine, isoleucine, valine, serine, alanine, phenylalanine, tyrosine, and glycine contributed the most to the PC1 and PC2 as observed by the intense red color in the contributions scale (Fig. 3, Supplementary Fig. 2).

**Figure 4:**
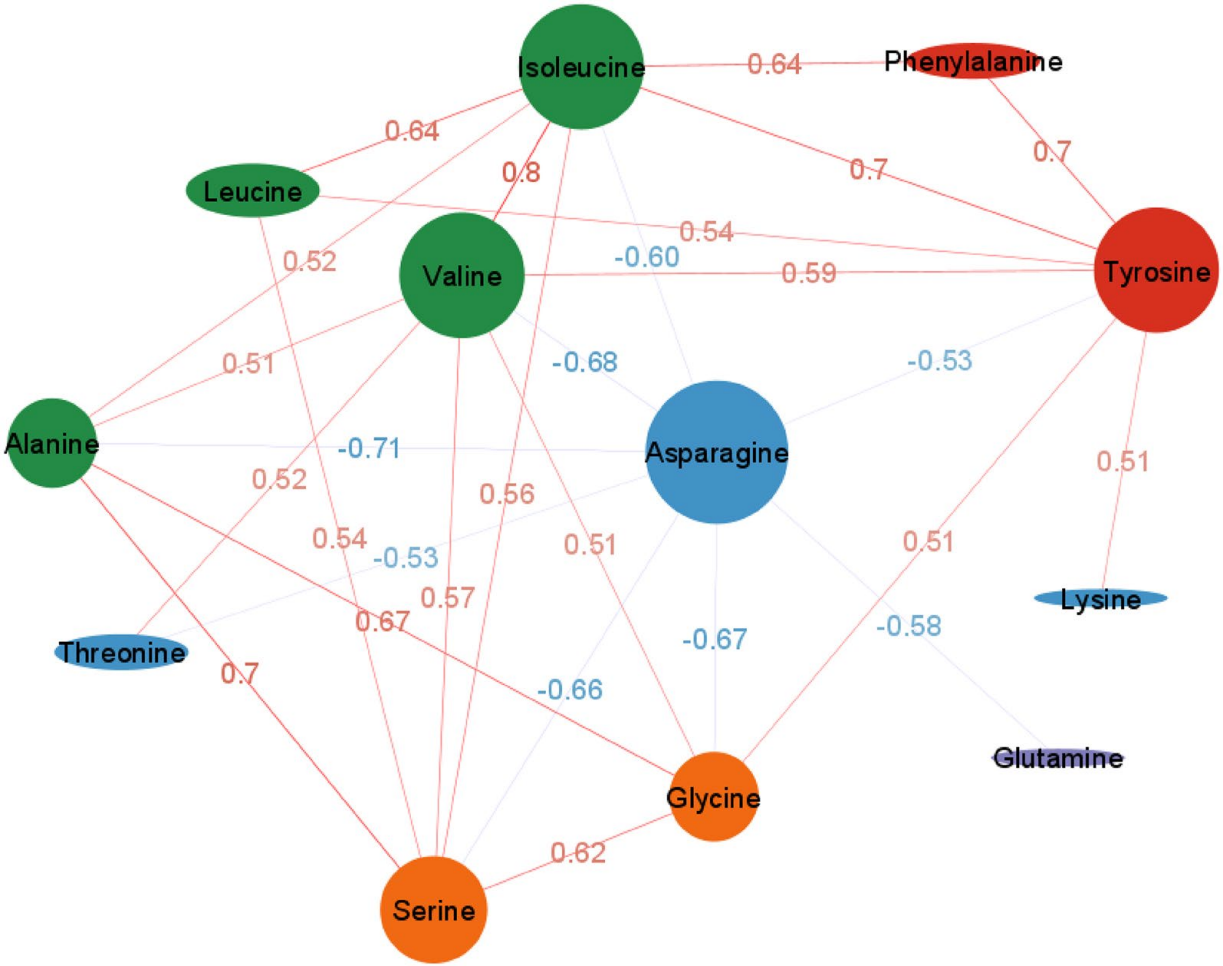
Correlation network of the free amino acid composition based on Pearson’s correlation matrix obtained based on the evaluation of 217 potato clones in Dalhart, Texas in 2019 and 2020, and Springlake, Texas in 2020. The correlation coefficient threshold was set at 0.5 with a *p*-value of less than 0.05.

The relationships among free amino acids in potato tubers were examined through a correlation network (Fig. 4). The correlation coefficient threshold for the network was set at 0.5, with *p*-values less than 0.05. The network consisted of 12 connected amino acids, and correlation coefficients were positive except with asparagine. Asparagine had the highest number of connections (nodal degree), followed by valine, tyrosine, valine, and serine, indicating its centrality to the network. The most abundant amino acid, glutamine, had a low level of connection. The connection between lysine, phenylalanine, and threonine was also poor. Valine had the highest positive and significant correlation with isoleucine (r = 0.80). Significant negative correlations were found between asparagine and alanine (r = − 0.71), valine (r = − 0.68), and glycine (r = − 0.67). Broad sense heritability values of the relative levels of free amino acids ranged from 0.58 to 0.89 for most amino acids except histidine, 0.15 (Table 1).

Average tuber weight per plant and average weight per tuber were significantly lower at Springlake, Texas compared to Dalhart, Texas. The correlations were examined and weak and/or moderate correlations were obtained. Interestingly, proline was negatively correlated with average tuber weight per plant and average weight per tuber (Table 2). Histidine was positively correlated with average tuber weight per plant and average weight per tuber (Table 2). There was no significant correlation between proline and average tuber number per plant. Also, no significant correlation was found between histidine and average tuber number per plant. The correlations of other amino acids with yield components were also examined. Negative correlation was observed for aspartic acid and proline with tuber weight (Supplementary Table 5). Likewise, positive correlations were observed for lysine, tyrosine, and methionine with tuber weight (Supplementary Table 5).

**Table 2:** Pearson correlations (r) between proline, histidine and yield related traits evaluated in Dalhart, Texas in 2019 and 2020, and Springlake, Texas in 2020.

	Proline	Histidine	Av. tuber weight per plant	Av. weight per tuber	Av. tuber no per plant
Proline	1.00				
Histidine	***−0.55***^†^	1.00			
Av. tuber weight per plant	− ***0.35***^†^	***0.35***^†^	1.00		
Av. weight per tuber	− ***0.30***^†^	***0.25***^†^	***0.57***^†^	1.00	
Av. tuber no per plant	0.06	0.02	***0.20***^†^	− ***0.60***^†^	1.00

^†^Significant at *p* < 0.001.

### Linkage disequilibrium (LD)

Using 10,106 SNPs for the full panel, pairwise LD was assessed using the squared-allele frequency correlations (r^2^), and LD was found to be low even at zero distance (Supplementary Fig. 3). The function LD.plot was used to illustrate the amount of LD in the panel. The 5–10 Mb window size seems appropriate to filter the output such that just the most significant marker inside a given window is returned based on the curve’s shape.

### Genome-wide association mapping—quantitative trait loci identification

**Figure 5:**
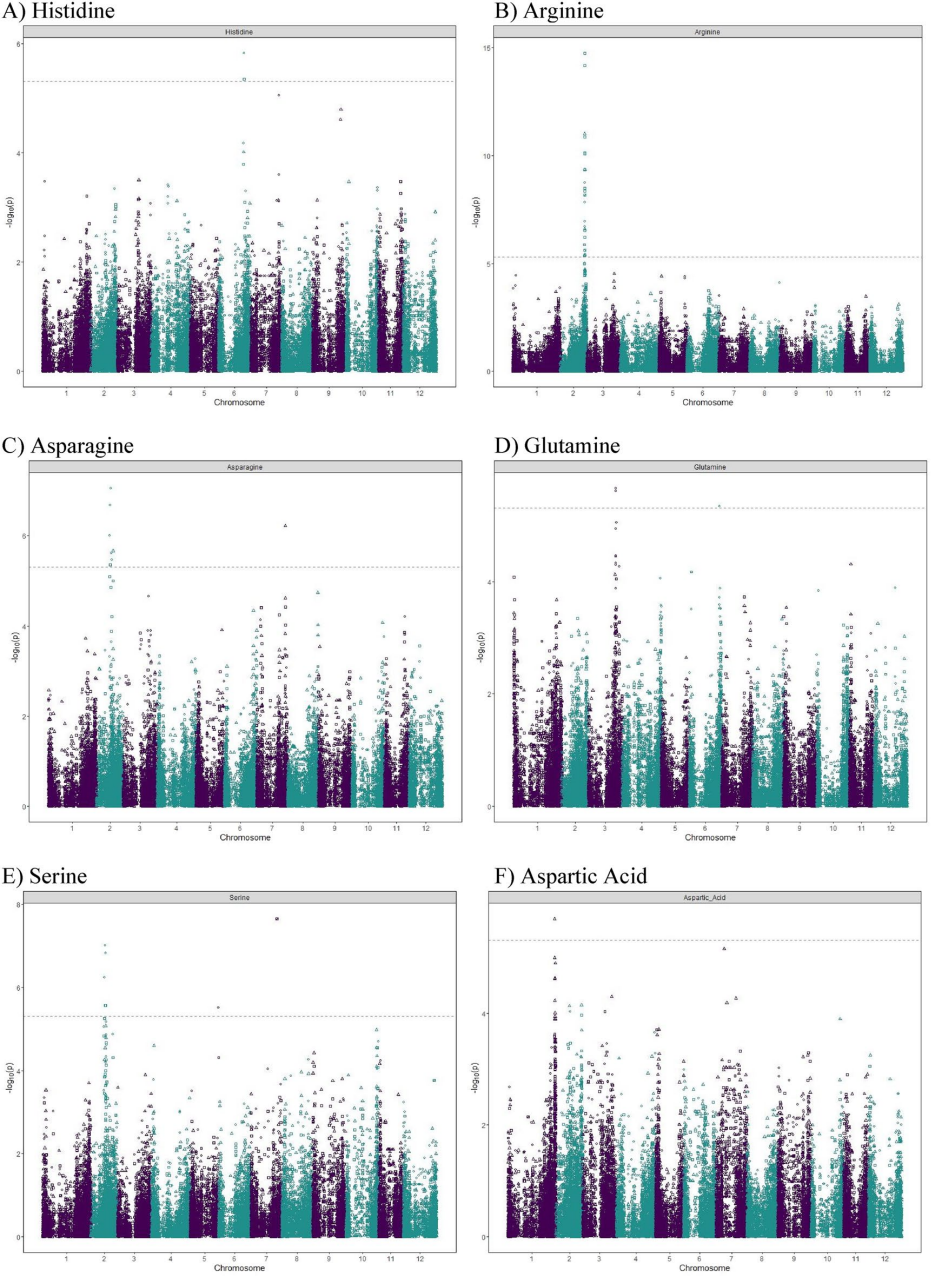

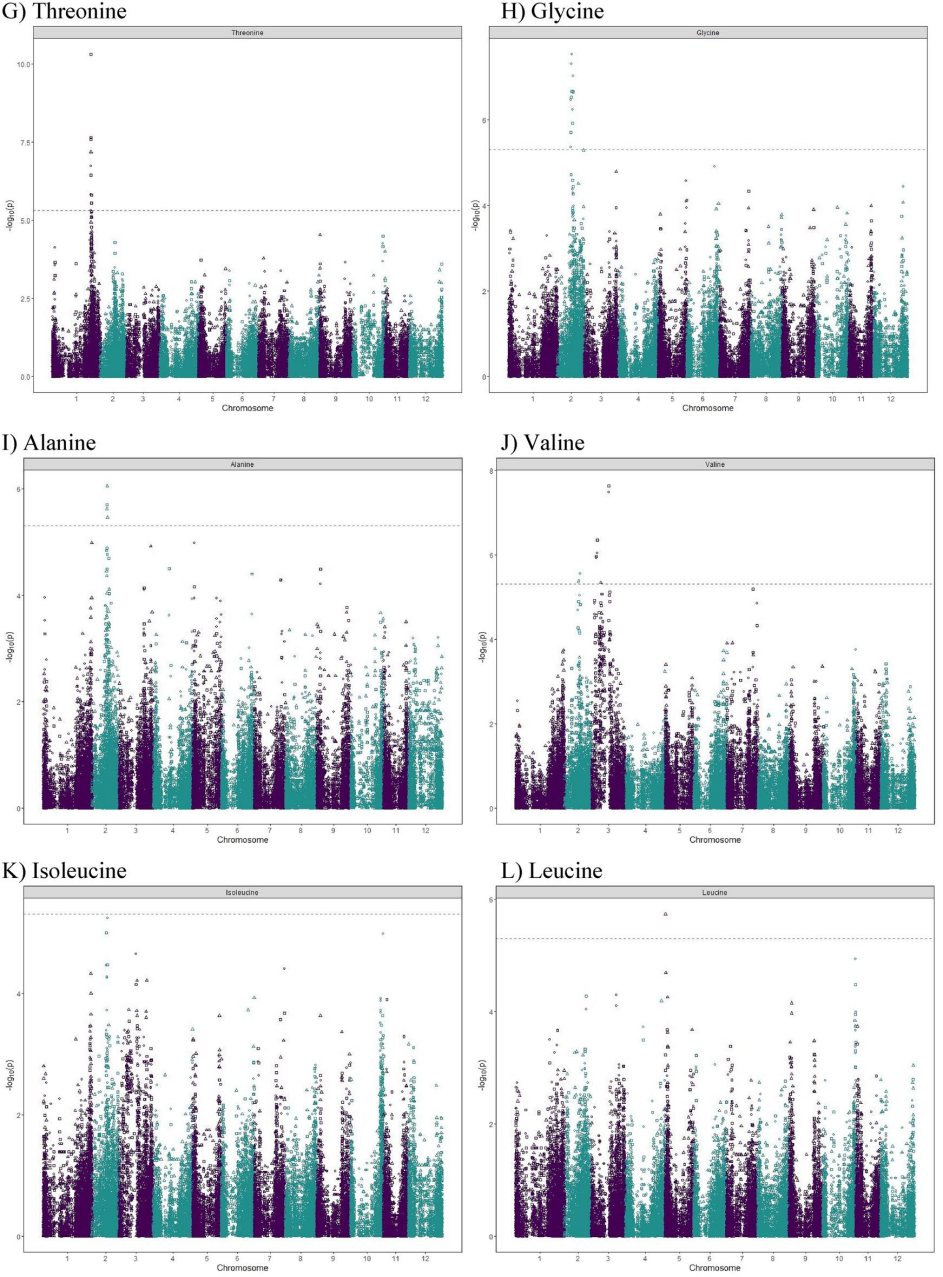

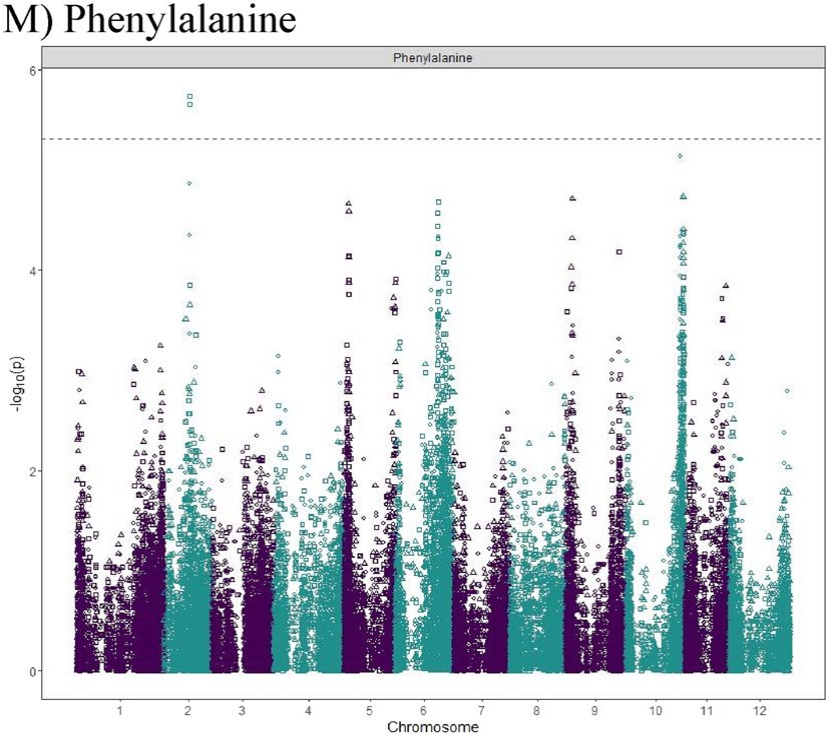
Manhattan plots displaying the marker–trait associations for Histidine (**A**), Arginine (**B**), Asparagine (**C**), Glutamine (**D**), Serine (**E**), Aspartic Acid (**F**), Threonine (**G**), Glycine (**H**), Alanine (**I**), Valine (**J**), Isoleucine (**K**), Leucine (**L**), Phenylalanine (**M**) by GWASpoly using the additive and dominant model in three combined environments. The horizontal axis indicates the chromosome number and the position of each SNP. The vertical axis indicates the negative logarithm of the *p*-value for each SNP. Each dot signifies an SNP. The broken line indicates the Bonferroni threshold level of 0.05.

We examined the inflation of the − log10(p) using quantile–quantile (QQ) plots of the observed vs. expected values under the null hypothesis shown in Supplementary Fig. 4. Strongly associated SNPs deviated from the plot’s diagonal at the upper-right end. A plot of the − log10(*p*-value) of the association statistic on the y-axis versus the chromosomal position of the SNP on the x-axis, commonly known as Manhattan plots, gave the visualization of the GWAS results (Fig. 5). Along the plot, regions with many highly linked SNPs in linkage disequilibrium emerged as the “Manhattan skyline”.

Allele dosage and different inheritance models were considered in GWASpoly^58^. The additive inheritance model identified nine SNPs associated with eight free amino acids. The simplex dominant models identified seven (1-dom ref) and eight (1-dom alt) SNPs associated with eight and nine free amino acids, respectively (Table 3). The duplex dominant models identified six (2-dom ref) and five (2-dom alt) SNPs associated with six and five free amino acids, respectively (Table 3).

**Table 3:** Significant marker-trit associations identified for the free amino acid samples obtained from three field experiments in Dalhart, Texas in 2019 and 2020, and Springlake, Texas in 2020.

Amino acid	Model	Threhold	SNP at QTL peak	Chr	Peak position (bp)	Score − log10(p)	Effect	R^2^ (%)
Histidine	additive	5.3	PotVar0070127	6	46,406,899	5.3	− 0.2	3.3
Histidine	1-dom-ref	5.1	PotVar0070127	6	46,406,899	5.8	− 0.2	3.6
Arginine	additive	5.3	solcap_snp_c1_12771	2	44,810,486	14.8	− 0.7	10.5
Arginine	1-dom-alt	5.0	solcap_snp_c2_43350	2	44,773,246	7.8	1.0	5.3
Arginine	1-dom-ref	5.1	solcap_snp_c1_12771	2	44,810,486	8.8	− 0.9	6.0
Arginine	2-dom-alt	5.2	PotVar0001749	2	45,037,953	5.4	0.8	3.4
Arginine	2-dom-ref	5.2	PotVar0001550	2	44,561,226	11.0	− 1.1	7.7
Asparagine	additive	5.3	solcap_snp_c2_52656	2	24,920,780	5.4	4.3	3.4
Asparagine	1-dom-alt	5.0	solcap_snp_c2_21654	2	25,560,378	7.1	6.4	4.5
Asparagine	1-dom-ref	5.1	PotVar0089318	2	26,584,883	5.6	− 5.3	3.6
Asparagine	2-dom-alt	5.2	PotVar0044416	7	52,317,483	6.2	8.1	4.0
Asparagine	2-dom-ref	5.2	solcap_snp_c1_13929	2	29,929,027	5.7	− 3.8	3.5
Glutamine	1-dom-alt	5.0	PotVar0041119	6	55,411,335	5.3	− 4.3	3.3
Glutamine	1-dom-ref	5.1	solcap_snp_c1_439	3	50,959,945	5.7	3.3	3.6
Serine	additive	5.3	PotVar0089318	2	26,584,883	5.6	0.4	2.2
Serine	additive	5.3	solcap_snp_c2_38787	7	47,875,892	7.7	0.7	3.4
Serine	1-dom-alt	5.0	solcap_snp_c2_21654	2	25,560,378	7.0	− 0.7	3.4
Serine	1-dom-alt	5.0	PotVar0034567	5	51,698,206	5.5	− 0.4	2.0
Serine	1-dom-ref	5.1	PotVar0089318	2	26,584,883	6.8	0.6	2.6
Serine	1-dom-ref	5.1	solcap_snp_c2_38787	7	47,875,892	7.7	0.7	2.9
Aspartic acid	2-dom-alt	5.2	solcap_snp_c2_53088	1	85,861,165	5.7	− 1.1	3.5
Threonine	additive	5.3	solcap_snp_c2_12087	1	71,176,726	10.3	− 0.3	7.1
Threonine	1-dom-alt	5.0	solcap_snp_c2_12092	1	71,205,164	5.8	0.3	3.7
Threonine	1-dom-ref	5.1	solcap_snp_c2_12087	1	71,176,726	6.7	− 0.4	4.4
Threonine	2-dom-ref	5.2	solcap_snp_c2_12087	1	71,176,726	7.2	− 0.6	4.7
Glycine	additive	5.3	solcap_snp_c2_21654	2	25,560,378	6.7	− 0.2	4.3
Glycine	1-dom-alt	5.0	solcap_snp_c2_21654	2	25,560,378	7.5	− 0.3	4.9
Glycine	1-dom-ref	5.1	PotVar0089318	2	26,584,883	6.2	0.3	4.0
Glycine	2-dom-ref	5.2	PotVar0002461	2	46,623,091	5.3	− 0.2	3.3
Alanine	additive	5.3	PotVar0089338	2	26,606,332	5.7	0.5	3.6
Alanine	2-dom-alt	5.2	PotVar0124308	2	27,089,538	6.1	− 1.2	3.8
Alanine	2-dom-ref	5.2	PotVar0124132	2	27,325,817	5.5	− 0.9	3.4
Valine	1-dom-alt	5.0	solcap_snp_c2_38935	2	27,860,245	5.6	− 1.3	1.6
Valine	1-dom-alt	5.0	solcap_snp_c2_5742	3	10,383,457	6.0	− 1.2	2.5
Valine	1-dom-ref	5.1	solcap_snp_c1_4775	3	31,056,417	7.5	1.5	1.2
Valine	2-dom-alt	5.2	solcap_snp_c2_36018	3	17,013,558	5.3	0.8	3.3
Isoleucine	1-dom-alt	5.0	solcap_snp_c2_38935	2	27,860,245	5.2	− 0.5	3.3
Leucine	2-dom-ref	5.2	PotVar0048910	5	349,077	5.7	− 0.7	3.6
Phenylalanine	additive	5.3	PotVar0117639	2	25,332,954	5.7	0.3	3.6
Phenylalanine	1-dom-ref	5.1	solcap_snp_c1_5010	10	55,210,592	5.1	0.3	3.2

A total of 33 QTL were significantly associated with 13 free amino acids on chromosomes 1, 2, 3, 5, 6, 7, and 10 (Table 3). However, no QTL were detected above the threshold for five amino acids, which are glutamic acid, proline, lysine, tyrosine, and methionine. A QTL on chromosome 6 was identified for histidine in the additive and the dominant model. GWAS analysis of arginine content identified four QTL in chromosome 2. Five QTL were identified for asparagine content in chromosomes 2 and 7. Two QTL were identified for glutamine content in chromosomes 3 and 6. Four QTL were identified for serine content in chromosomes 2, 5, and 7. For aspartic acid content, the only QTL identified was in chromosome 1. For threonine content, two QTL were identified in chromosome 1. Three QTL were identified for glycine content in chromosome 2. Three QTL were identified for alanine content in chromosome 2. Four QTL were identified for valine content in chromosomes 2 and 3. One QTL was identified for isoleucine content in chromosome 2. A QTL was observed in chromosome 5 for leucine content, which explained 3.6% of the phenotypic variance. Two QTL were identified for phenylalanine content on chromosomes 2 and 10 under additive and dominant genetic models, respectively. QTL co-localization was discovered for a few amino acids regardless of the inheritance model tested. Asparagine, glycine, and serine showed two colocalizing QTL on chromosome 2 (SNP peak positions at 25.6 Mb and 26.6 Mb), and isoleucine and valine shared a QTL in another region of chromosome 2 (SNPs peak position at 27.9 Mb). The position of the QTL, SNP at the peak and the phenotypic variance explained by each QTL are reported in Table 3.

The genes located in the 100 kbp (kilobase pair) flanking genomic region of each peak SNP were retrieved from the potato reference genome (Supplementary Table 6). The selection of a 100 kbp interval is a commonly used size range when investigating candidate genes. Gene annotation of DM version 4.03 and UniProt (www.uniprot.org) were used to evaluate these candidate genes’ putative function. Several of the genes (around 150 candidate genes) had unknown functions (Supplementary Table 6). However, a few potential genes directly linked to primary metabolic pathways were found in our investigation. A proenzyme, S-adenosylmethionine decarboxylase proenzyme (SAMDC) (PGSC0003DMG400010051), was identified as part of the arginine metabolism. Another potential gene, proline oxidase/dehydrogenase (PGSC0003DMG400010050), was found for proline metabolism (Supplementary Table 6). Given the degree of linkage disequilibrium in potatoes, it cannot be ruled out that genes regulating amino acids could be located megabases away from important SNPs.

### Genomic selection – genomic-estimated breeding values for traits

**Figure 6:**
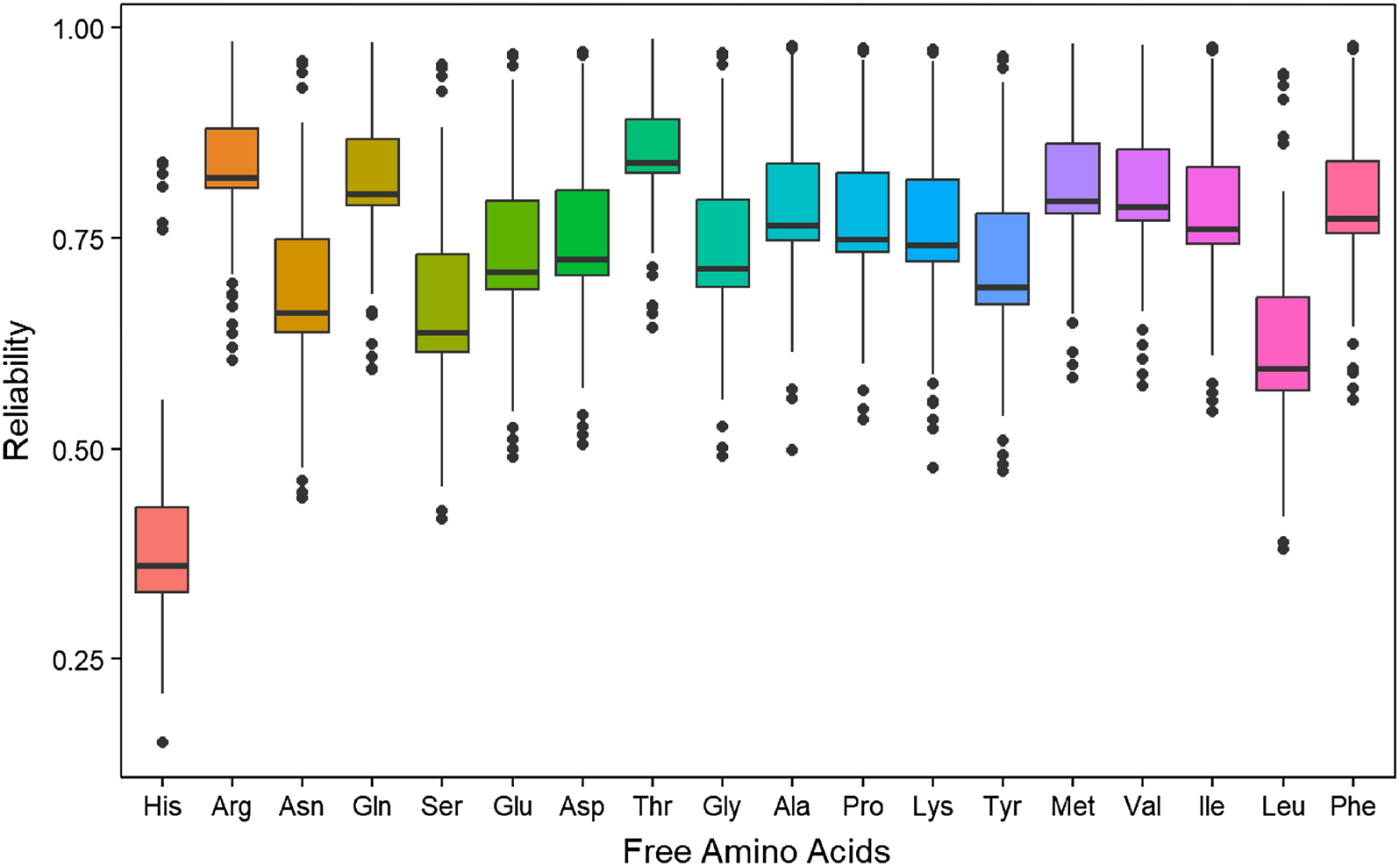
Box plot of the predicted reliabilities (the squared correlation between the true and predicted values) for free amino acids content (His = histidine, Arg = arginine, Asn = asparagine, Gln = glutamine, Ser = serine, Glu = glutamic acid, Asp = aspartic acid, Thr = threonine, Gly = glycine, Ala = alanine, Pro = proline, Lys = lysine, Tyr = tyrosine, Met = methionine, Val = valine, Ile = isoleucine, Leu = leucine, Phe = phenylalanine) in 217 potato clones.

Genomic estimated breeding values (GEBVs) can help guide parental selection and the advancement of superior clones in breeding programs. Box plot of the predicted reliabilities (the squared correlation between the true and predicted values) showed that most of the predicted reliabilities are higher than 0.5 for most of the free amino acids content except histidine (Fig. 6). The GEBVs (Supplementary Table 7) showed that clone TX12474-1P/R ranked topmost for histidine. NDTX060700C-1W ranked topmost for asparagine and aspartic acid. Considering the GEBVs, the topmost was BTX1544-2W/Y for arginine, NDTX050169-1R for glutamine, and COTX00104-7R for serine content. BTX1544-2W/Y came out on top for arginine, whereas NDTX050169-1R came out on top for glutamine. COTX00104-7R had the highest GEBV for serine content, and AOTX97213-1Ru had the highest GEBV for glutamic acid. ATTX96746-1R came out on top for threonine, and Krantz came on top for glycine. COTX05211-4R ranked topmost for alanine, and NDTX081648CB-4W ranked topmost for proline. COTX04015-3W/Y had the highest GEBVs for lysine. COTX05261-1R/Y ranked topmost for tyrosine, and ATX9130-1Ru ranked topmost for methionine. Based on the GEBVs, ATTX98500-3P/Y came on top for valine. The topmost clone for isoleucine was ATTX98510-1R/Y, leucine was ATTX03516-2R, and phenylalanine was ATTX10265-4R/Y.

### Weighted standardized multitrait selection indexes

Chipping clones with all other amino acids rich but with low asparagine was found using weighted normalized multitrait selection indexes (Supplementary Table 8). Three clones (NDTX059828-2W, ATTX95490-2W and AOTX95309-2W) were found to have the highest rankings (Z-score > 2) among the chipping potatoes (Supplementary Table 8).

## Discussion

This study uncovered the natural variation for various free amino acids in a potato diversity panel from a potato breeding program in the USA^54^ and linked phenotypic variation for several traits to genetic markers to understand the genetic basis of the traits and obtain genomic-estimated breeding values to improve breeding efficiencies. The panel of potato clones used included reference varieties for various market groups and advanced selections of cultivated tetraploid potatoes from the Texas A&M Potato Breeding Program, developed over 40 years. The outcome of this research should guide breeding efforts in potatoes to improve health, nutritional, and processing qualities.

Significant differences were found between tetraploid potato clones for the relative quantity of all 19 free amino acids evaluated. Variations in the amounts of free amino acids were found in potato tubers from different growing locations and years^70^. Asparagine and glutamine were the most abundant amino acids, with average relative compositions of 33.0% and 21.0%, respectively in our potato diversity panel. Asparagine levels showed great variation in the panel, from 21.0 to 55.0%. The most prevalent amino acids in potato tubers were asparagine and glutamine, which together made up to 90.0% of the total free amino acid content^10^. In another study, asparagine accounted for the greatest proportion of free amino acids (up to 30.0–36.0%)^71^. Asparagine and reducing sugars form acrylamide, a carcinogenic chemical that is a concern, especially in processed potato products^28^. Both asparagine and reducing sugars should be reduced to decrease acrylamide content in processing potatoes^42^. According to this study, targeted breeding efforts would be required to reduce the asparagine content of conventionally bred potatoes to the range required for acrylamide mitigation. Since potatoes had never been bred for low tuber asparagine content, the effectiveness of this strategy was questionable^42^. Our study found that the chipping (processing) market group had significantly higher asparagine content in raw tubers than other market groups (Supplementary Table 4). A previous study showed a negative correlation between tuber sucrose and amino acid content^45^. It reported that tubers with a reduced sucrose transporter expression displayed significant increases in alanine, arginine, asparagine, glutamine, glycine, serine, threonine, and valine^45^. In selecting chip potatoes with low-reducing sugars and cold-sweetening resistance, these breeding efforts may have favored indirect selection for higher accumulation of free amino acids including asparagine, but additional research is needed to confirm this relationship. Some research has shown that asparagine content in potatoes is larger and less variable than glucose and fructose, which helps to explain why reducing asparagine could be challenging^22^. On the other hand, researchers have shown that specific genotypes with low asparagine contents can be exploited in breeding efforts to lower tuber asparagine content^41,72^.

Ideally, breeders should aim for the identification and development of processing potato cultivars with low free-asparagine and reducing sugar as desirable characteristics for processing purposes. Recently, in a processing cultivar, FL-1533, a distinctive metabolite combination was found to have the least amounts of reducing sugar and asparagine compared to the other cultivars^73^. The availability of such germplasm would make it possible to introduce the low sugar and low asparagine traits into varieties acceptable for broad use in the food industry. The implications for low asparagine on potato yield, disease suceptibilty, starch properties etc. are limited but it would be worth exploring in the future. However, previous studies indicated that certain amino acids affect the physicochemical properties of potato starch^74^. Charge-carrying amino acids (lysine, arginine, aspartic acid, and glutamic acid) can potentially alter the physical and chemical characteristics of starch-based products derived from potatoes^74^. Increased amounts of charge-carrying amino acids during heating can lead to notable improvements in their nutritional qualities, achieved by reducing the swelling power, solubility, light transmittance, and gel strength of the potato starch^74^. Another study found that amino acids, particularly glutamic acid and aspartic acid, can affect the gelatinization, pasting, and rheological properties of potato starches^75^, potentially resulting in effects on food texture and mouthfeel, and influencing consumer acceptance.

Another essential amino acid, methionine, was significantly higher in russets and lowest in purples (Supplementary Table 4). The potato industry is interested in boosting the tuber methionine content because of the related nutritional and fragrance benefits^32^. Even though methionine is an important amino acid, no related QTL were found in the analysis. Increasing the sample size and studying more diverse populations could help capture a broader range of genetic variation and improve the chances of identifying relevant associations. If unearthing natural variation becomes a limitation, the biotechnological interventions to manipulate methionine metabolism is a parallel strategy. Lysine’s average relative composition in the diversity panel was 3.3%, and the clone with the highest proportion of lysine (5.6%) was COTX03134-1Y/W. Potato is a good source of lysine and thus can be used as a supplement to cereals deficient in this amino acid^76^.

Despite using similar production practices across the Texas potato field locations, the analysis of variance indicated significant environmental effects when comparing locations for histidine and proline content. On average, tubers produced at the Springlake location have lower histidine and higher proline content than those produced in Dalhart. Quantitative differences in the amino acid content between varieties based on location were previously reported^77^. Proline was the amino acid that varied the greatest between the two regions which differed in rainfall and humidity^77^. The Springlake and Dalhart locations differ for altitude, latitude, and longitude; day/night temperatures, photoperiod, radiation, type of soil, and biotic and abiotic stresses. The Springlake location regularly has higher night temperatures (often > 20 °C near harvest), resulting in lower marketable yield and more external and internal tuber defects as result of having more heat stress than the Dalhart location. Experiments under controlled greenhouse conditions showed that heat stress-induced chemical changes in potato tubers^78^. Proline is crucial for plant growth and differentiation throughout the whole life cycle and also serves as a superior osmolyte and also performs three other crucial functions under stress, including metal chelation, antioxidant defense, and signaling^79^. To protect plants from heat stress, proline functions as a signaling molecule^80^. Our findings indicated that proline levels in tubers produced at Springlake, a location suffering abiotic stresses (mainly heat stress), were significantly higher (129% higher) than a more favorable location (Dalhart) that, despite having hot days during the day, has more cool nights that favor the accumulation of starch in tubers and less external and internal tuber defects. Proline is a precursor for other downstream metabolites, including polyamines and secondary metabolites and thus may affect tuber quality. Proline can be produced from arginine and glutamine/glutamate in the majority of animals, including humans and pigs, although rates of endogenous synthesis are insufficient for newborns, fish, and birds^81^, thus consuming proline-rich produce should be beneficial to contribute to the production of proteins in general, and collagen in particular, and thus help in the formation of skin, cartilages, and bones. Likewise, histidine is an essential amino acid for plant growth and development^82^. Our findings showed lower level of histidine in tubers in the stressful site Springlake. This result conflicts with the amount of free histidine present in canola cultivars, as histidine, serine, and cysteine were found to rise in the xylem sap of various canola cultivars during nickel toxicity^83^. Further research is needed to fully understand if stress induces low histidine in tubers.

In our study, years did not significantly affect the content of free amino acids in tubers whereas location had a significant effect in histidine and proline. While breeding for high (or low) amino acids, multilocation evaluations would be more important than multiyear evaluations.

In this study, branched-chain amino acids (isoleucine and valine) displayed high correlation values (r = 0.8). Aromatic amino acids (i.e., tyrosine and phenylalanine) also highly correlated (r = 0.7). High correlations were anticipated due to linked pathways. Previous studies in potatoes reported r > 0.7 between branched-chain amino acids^46^ and r = 0.7 between serine and glycine^84^. However, in our research, unexpected correlations were also observed between amino acids that are not closely linked biosynthetically (e.g., phenylalanine and isoleucine, alanine and serine, and alanine and glycine). In previous metabolic profile studies of potato tubers, unexpected amino acid associations were also discovered^46,84,85^. Asparagine and other amino acids were found to have significant negative correlations, suggesting that decreasing asparagine would probably increase the other amino acids involved in the correlations and vice versa.

In this study, proline content was negatively correlated and histidine content was positively correlated with yield components but the correlations were weak. The weaker correlations would likely make them unsuitable to use as markers for yield potential. The relationship between proline and histidine content and potato yield components are limited. However, the relationship may vary depending on several variables, including the type and degree of stress, the potato cultivar, and other environmental factors. In wheat, grain proline content during a drought was linked to greater thousand grain weight loss and does not seem to act positively in terms of productivity^86^.

Thanks to recent developments in high-throughput metabolic profiling and sequencing technologies, GWAS has been used as a powerful strategy to unveil crop metabolism’s genetic and biochemical underpinnings. GWASpoly is suited for conducting GWAS in polyploids as it investigates different inheritance models^58^. For example, in this study, nine SNPs were shown to be linked to eight free amino acids with the additive inheritance model. Each additional copy of the allele can boost the trait’s output. Using GWASpoly, we identified 33 QTL associated with 13 free amino acids in this study. In general, only a small portion (< 10%) of the phenotypic variance could be accounted by each QTL, except for arginine (10.5%). Co-localization of QTL for free amino acids was observed in a few cases independently of the model of inheritance investigated. It is not unusual to see colocalized QTL for biochemically related amino acids. For example, in the present study, isoleucine and valine had the strongest, most positive, and most significant correlation (r = 0.80), and shared a QTL in chromosome 2 (SNPs peak position at 27.9 Mb). Previous research on potatoes has shown a high interdependence of biochemically related amino acids^46^. A shared QTL could come from closely linked independent regulators or a common upstream regulator^46^ for amino acids that are not biochemically related. QTL linked to various amino acids can potentially improve multiple amino acid content in potatoes simultaneously, and they deserve further investigation.

By assigning potential candidate genes to GWAS outcomes based on metabolites, it is postulated that the candidate loci directly or indirectly encode intermediate products that would biochemically catalyze, modify, translocate, or regulate the associated metabolites- which thus warrants systemic evaluation to link them to end phenotypes. The success of GWAS in mapping genes is limited in its resolution due to the high level of LD in the breeding germplasm, annotation of the reference genome, and the number of high-quality SNPs. This method provides only indirect evidence for the association of the genomic region with individual amino acids. We anticipate continuing additional research to validate the functional role of these genes in using model species or transgenic approaches. Nevertheless, this study has established a foundation to analyze identified loci and their evolutionary significance in amino acid metabolism in potatoes. In our study, the genes within 100 kbp window size were identified according to the positions of the closest flanking significant SNPs. Several independent studies have used genomic space within ± 100 kb of each peak SNP proximity to identify candidate genes associated with varied traits using association mapping in potato^87,88^. Expanding the search interval to 100 kbp increases the likelihood of capturing genetic variants in LD with the causal variant, improving the chances of identifying relevant candidate genes. In some instances, narrower or broader intervals may be considered, depending on the context and objectives of the study.

Our analysis did uncover a few candidate genes that are directly connected to major metabolic processes. Based on the KEGG pathway (SOT 102,589,057), S-adenosylmethionine decarboxylase proenzyme (SAMDC)—PGSC0003DMG400010051 was mapped to arginine metabolism. S-adenosylmethionine decarboxylase synthesizes polyamines putrescine, spermidine, and spermine across species^89^. Arginine is the ornithine precursor, the substrate needed for polyamines biosynthesis. Ornithine decarboxylase converts ornithine to putrescine, which is further converted to spermidine and spermine by consuming decarboxylated S-adenosylmethionine^90^. Transgenic potato plants expressing antisense SAMDC showed stunted phenotypes, reduced levels of SAMDC transcripts and enzyme activity, and polyamine content, suggesting the significance of polyamines in the tuber formation^91^. Additionally, GWAS of sorghum grain qualities identified S-adenosylmethionine decarboxylase proenzyme mapped to arginine, cysteine, methionine, proline, and starch metabolism^92^. It will be intriguing to perform additional genetic characterization to understand the role of this gene in polyamine synthesis or, like sorghum, its relevance in starch synthesis in potatoes. Another candidate gene, Proline oxidase/dehydrogenase (PGSC0003DMG400010050), catalyzes proline oxidation to Δ^1^—Pyrroline—5-Carboxylate Synthetase (P5C) in the mitochondria. Proline and arginine metabolic pathways are interconnected^93^ as the second and last step in proline catabolism, P5C to glutamate is shared with arginine catabolism^94^. Arginine can serve as a precursor and product of ornithine during the urea cycle via arginase, converting arginine to ornithine^95^. Our study identified limited candidate genes directly associated with primary metabolic pathways, which is plausible given the complexity of their biosynthesis and catabolism involving several intermediates, their transport, and feedback regulations. Detailed characterization of identified genes using functional approaches such as cloning and heterologous expression of genes in model species, tissue-specific knocking out genes using RNAi, antisense methods, or CRISPR-Cas would help understand their relevance in amino acid metabolism in potatoes.

GEBV was obtained for all members of the diversity panel (217 clones). GEBVs will allow faster and more effective selection of superior parents and/or the advancement of better clones. The predicted reliabilities in this study were encouraging, except for histidine. Prediction ability is influenced by the genetic make-up of the traits under investigation, the training population, and how similar the training population is to the validation population. Recently, software programs like StageWise^96^ are being developed for polyploid crops to manage the numerous experimental designs, heritabilities, and spatial models for nongenetic variation. Individual GEBVs will likely not be used for selection since there are many important traits to consider in selection. To use GEBVs for selection, GEBVs for several traits might be merged into an overall index. In this study we utilized the standardized indexes for the chipping clones. Not all traits will have the same importance, and not all traits are measured using the same units or scales; thus, standardized scores and relative weight should be assigned to each trait to facilitate the selection of superior and inferior genotypes^97^. Market demands, industry needs, and several other considerations drive the relative importance of traits. Using GEBVs to select potatoes with particular concentrations could significantly improve potatoes’ health and nutritional value. GEBVs for traits such as yield, quality, disease/pest resistance, and tolerance to abiotic stresses should be part of the combined selection index to maximize the chances of selecting the best clones and make selection more efficient. For practical applications, GEBVs should be considered in the context of market groups and selection criteria.

## Materials and methods

### Plant materials and experimentation

A collection of 217 tetraploid potato clones was used for this study. The diversity panel represents varieties and advanced genotypes selected over four decades by the Texas A&M Potato Breeding Program. Details of the potato diversity panel used were described earlier^54^. The clones included fresh and processing market classes with variations in tuber shape, skin type, skin and flesh color, biotic and abiotic stress tolerance, and quality traits^54^.

Three field experiments were conducted at two locations in Texas in commercial potato grower fields. In 2019, the diversity panel was planted in Dalhart (35°58′ N, 102°44′ W), and in 2020 the clones were planted in Dalhart and Springlake (34°6′ N, 102°19′ W) in a 12-hill plot with two replications. Dalhart is approx. 268 km North of Springlake and 92 m higher. Both Texas locations experience high-temperature stress during the growing season, however, Dalhart yields often duplicate the yields in Springlake (https://potato.tamu.edu/reports/), likely due to cooler nights at Dalhart. Trials were planted in early May in Dalhart, Texas, and harvested in early September. At Springlake, trials were planted in late March to early April and harvested in early July. Vine desiccation was done 2–3 weeks before harvesting the trials. Standard production practices were used. Details about the field trials, including environmental conditions, spacing, irrigation, fertilization, weed, and insect control, can be obtained online for the corresponding years and locations from the 2019 and 2020 Texas A&M Potato Breeding Program reports (https://potato.tamu.edu/reports/).

### DNA extraction and genotyping

Genomic DNA was extracted from 50 to 80 mg of fresh young potato leaves from tissue culture plantlets using the DNeasy Plant Pro Kit (Qiagen, Valencia, CA, USA) as described earlier^54^. Samples were analyzed using the Infinium 22 K V3 Potato Array on the Illumina iScan (Illumina Inc., San Diego, CA, USA). 10,106 polymorphic SNPs were selected for analysis after filtering the marker dataset for minor allele frequency and polymorphism, as previously described^54^.

### Measurements of free amino acid content

Three tubers per plot were randomly selected and chopped into small cubes (4 mm per side). About 15 g of freshly chopped samples were collected into 50 mL tubes. Samples were stored in −80 C freezer for a few days before freeze-drying them using a Labconco FreeZone 6-L Freeze-dry System (Labconco, Kansas City, MO, USA) at a collector temperature of − 50 °C for 120 h at 22 Pa. Ceramic grinding cylinders (0.95 cm × 2.22 cm) with angle-cut ends (SPEX SamplePrep, Metuchen, NJ, USA) were used for homogenizing the dry tuber sample in 50 mL centrifuge tubes using SPEX Sample Prep 1600 MiniG tissue homogenizer (SPEX SamplePrep, Metuchen, NJ, USA) for 2 min set at a speed of 1500 strokes per minute. Powdered samples were stored at room temperature in tightly closed Minigrip® Red Line zip bags (Minigrip, Alpharetta, GA) until use.

Free amino acids from samples of potato tubers were extracted from two biological replications per environment (location-year combination) and three technical replicates per biological replication using an established protocol^98,99^. The Kairos™ Amino Acid Kit was used for amino acid calibration (Waters Corp., Milford, MA, USA). Waters Acquity UPLC H-class system with a Waters Xevo TQ mass spectrometer with an electrospray ionization (ESI) probe was used to detect amino acids. Each amino acid’s multiple reaction monitoring (MRM) transition, collision energy values, and cone voltage were optimized using IntelliStart software (Waters Corp., Milford, MA, USA). For instrument monitoring and data capture, Water’s MassLynxTM software was used. The TargetLynxTM Application Manager (Waters Corp., Milford, MA, USA) was employed for data integration, calibration curves, and amino acid quantification. The relative composition of free amino acid levels (each as a percentage of total free amino acids, e.g., alanine /total) was determined.

### Statistical analyses

Descriptive statistics (means, minimums, and maximums) were calculated for free amino acids using the software package META-R^100^. Phenotypic distributions for the traits were generated and normality tests were conducted by the Shapiro–Wilk W-Test^69^ using JMP Pro 16® statistical software (SAS Institute, Cary, NC). The free amino acids were separated into groups using a hierarchical clustering analysis using Euclidean distance metric and Ward’s minimal variance method. The effect of location, years, and environment (considered as location-year combination: Dalhart 2019, Dalhart 2020, and Springlake 2020), clone (217 clones), and the corresponding individual amino acid content of raw potato tubers were examined using analysis of variance using a mixed model in JMP Pro 16. Clones were considered fixed effects, while environment (location-year combination), replication within environments, and interactions were considered random.

Similarly, an analysis of variance was used to determine the effect of location (two field sites) on the traits (Dalhart and Springlake field trials conducted in 2020 were used). In this case, clones and locations were treated as fixed effects. Data from 2 years (2019, 2020) from one location (Dalhart) were used to ascertain the impact of years on the traits evaluated. Pearson’s correlation coefficients (r) were obtained, and a correlation network was built to examine and illustrate the relationships among free amino acids. A correlation coefficient threshold was set at 0.5 with a *p*-value of less than 0.05 to visualize the stronger correlations between free amino acids. A significant correlation matrix was generated using the R program (version 4.1.2), and the network visualization was performed with Cytoscape 3.9.1^101^, using a previously reported method^102^. Also, principal component analysis (PCA) was completed using the function prcomp in the stats package and plotted using the function ggbiplot^103^ and factoextra package (https://github.com/kassambara/factoextra). Confidence ellipses were included, representing 95% confidence intervals around the centroid value of each data cluster. The function fviz_cos2 was used to visualise the quality of representation (cos2) of the variables in the principal components. Pearson correlations (r) between selected amino acid and yield-related traits^104^ evaluated in Texas (Dalhart in 2019 and 2020, and Springlake in 2020) were also calculated.

### Genome-wide association analysis (GWAS)

Association analysis was performed for free amino acids with 10,116 SNPs using the GWASpoly Version 2 package in R^58^. The leave-one-chromosome-out (LOCO) method^105^ was used to account for population structure. For each trait, additive and dominant genetic models were tested. A LOD threshold for each trait, corresponding to a genome-wide false-positive rate of 5%, was established based on a Bonferroni test. Manhattan plots were generated using the GWASpoly package. GWASpoly estimated the proportion of phenotypic variance explained by significant SNPs at the peak of each QTL. Contextual sequences of SNPs at the peak of QTL were used in BLAST searches of DM1-3 pseudomolecules (Version 4.03) in the SpudDB database (http://solanaceae.plantbiology.msu.edu/) to identify putative candidate genes located in the 100 kbp (kilobase pair) flanking genomic region.

### Genomics selection (GS)—Genomic-estimated breeding values

Genetic variance partitioning and genome-wide prediction were performed using allele dosage information^96,97,104,106^. Standard errors and the best linear unbiased predictors (BLUP) were calculated using standard methods. For a generic random vector **u**, BLUP $\left[\mathbf{u}\right]=\widehat{\mathbf{u}}=\mathbf{C}\mathbf{P}\mathbf{y}$, where $\mathbf{C}=\mathrm{cov}[\mathbf{u},\mathbf{y}]$, $\mathbf{P}={\mathbf{V}}^{-1}-{\mathbf{V}}^{-1}\mathbf{X}{\left({\mathbf{X}}^{\mathrm{^{\prime}}}{\mathbf{V}}^{-1}\mathbf{X}\right)}^{-1}{\mathbf{X}}^{\boldsymbol{^{\prime}}}{\mathbf{V}}^{-1}$, **X** is the incidence matrix for fixed effects, and **V** is the variance–covariance matrix of the response variable^107^. The reliability of ${\widehat{u}}_{i}$ is represented by the squared correlation with the true value which equals ${\mathrm{r}}_{\mathrm{i}}^{2}=\frac{\mathrm{var}\left[{\widehat{\mathrm{u}}}_{\mathrm{i}}\right]}{\mathrm{var}\left[{\mathrm{u}}_{\mathrm{i}}\right]},$ and $\mathrm{var}\left[\widehat{\mathbf{u}}\right]=\mathbf{C}\mathbf{P}{\mathbf{C}}^{\mathbf{^{\prime}}}$.

### Weighted standardized multitrait selection indexes

Standardized multi-trait selection indexes were calculated for all amino acids in the chipping class based on the Z values of GEBVs^97^. The weighted multi-trait selection index for each clone was calculated assigning positive weight for all amino acids except asparagine. Asparagine was assigned a negative weight because lower values of asparagine are preferred by the processing industry.

### Supplementary Information

Supplementary Figure 1.

Supplementary Figure 2.

Supplementary Figure 3.

Supplementary Figure 4.

Supplementary Table 1.

Supplementary Table 2.

Supplementary Table 3.

Supplementary Table 4.

Supplementary Table 5.

Supplementary Table 6.

Supplementary Table 7.

Supplementary Table 8.

## Data Availability

The genotype datasets used during the current study are available in our previous article in Scientific Reports, https://www.nature.com/articles/s41598-021-87284-x#additional-information. All other data generated or analyzed during this study are included in this published article.
