# Supplementary material for: Genetic architecture of tuber-bound free amino acids in potato and effect of growing environment on the amino acid content
Source: Sci Rep. 2023 Aug 25;13:13940. doi: 10.1038/s41598-023-40880-5 (PMC10457394; doi:10.1038/s41598-023-40880-5)
Supplement: Supplementary file 1 — Supplementary Figure 1. [file 41598_2023_40880_MOESM1_ESM.docx]

**Supplementary Figures**


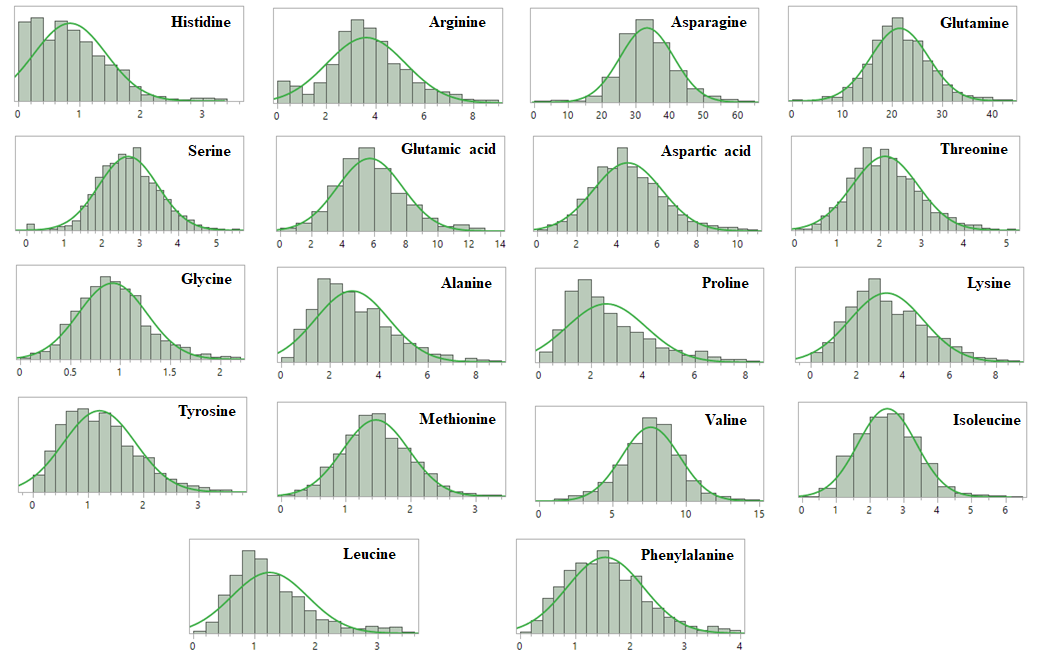


W = 0.983 P = 0.012

W = 0.987 P = 0.043

W = 0.991 P = 0.178

W = 0.973 P = 0.0004

W = 0.990 P = 0.149

W = 0.993 P = 0.456

W = 0.995 P = 0.689

W = 0.973 P = 0.0004

W = 0.945 P = <.0001

W = 0.995 P = 0.724

W = 0.997 P = 0.950

W = 0.979 P = 0.003

W = 0.994 P = 0.558

W = 0.993 P = 0.401

W = 0.995 P = 0.714

W = 0.973 P = 0.0004

W = 0.957 P = <.0001

W = 0.985 P = 0.029

**Supplementary Figure 1:** Distributions of percent free amino acids across 217 potato clones evaluated in Dalhart, Texas in 2019 and 2020, and Springlake, Texas in 2020. Both the test statistic (W) and associated p value from a Shapiro–Wilk normality test are shown.
