# Supplementary material for: Genetic architecture of tuber-bound free amino acids in potato and effect of growing environment on the amino acid content
Source: Sci Rep. 2023 Aug 25;13:13940. doi: 10.1038/s41598-023-40880-5 (PMC10457394; doi:10.1038/s41598-023-40880-5)
Supplement: Supplementary file 2 — Supplementary Figure 2. [file 41598_2023_40880_MOESM2_ESM.docx]

**Supplementary Figures**


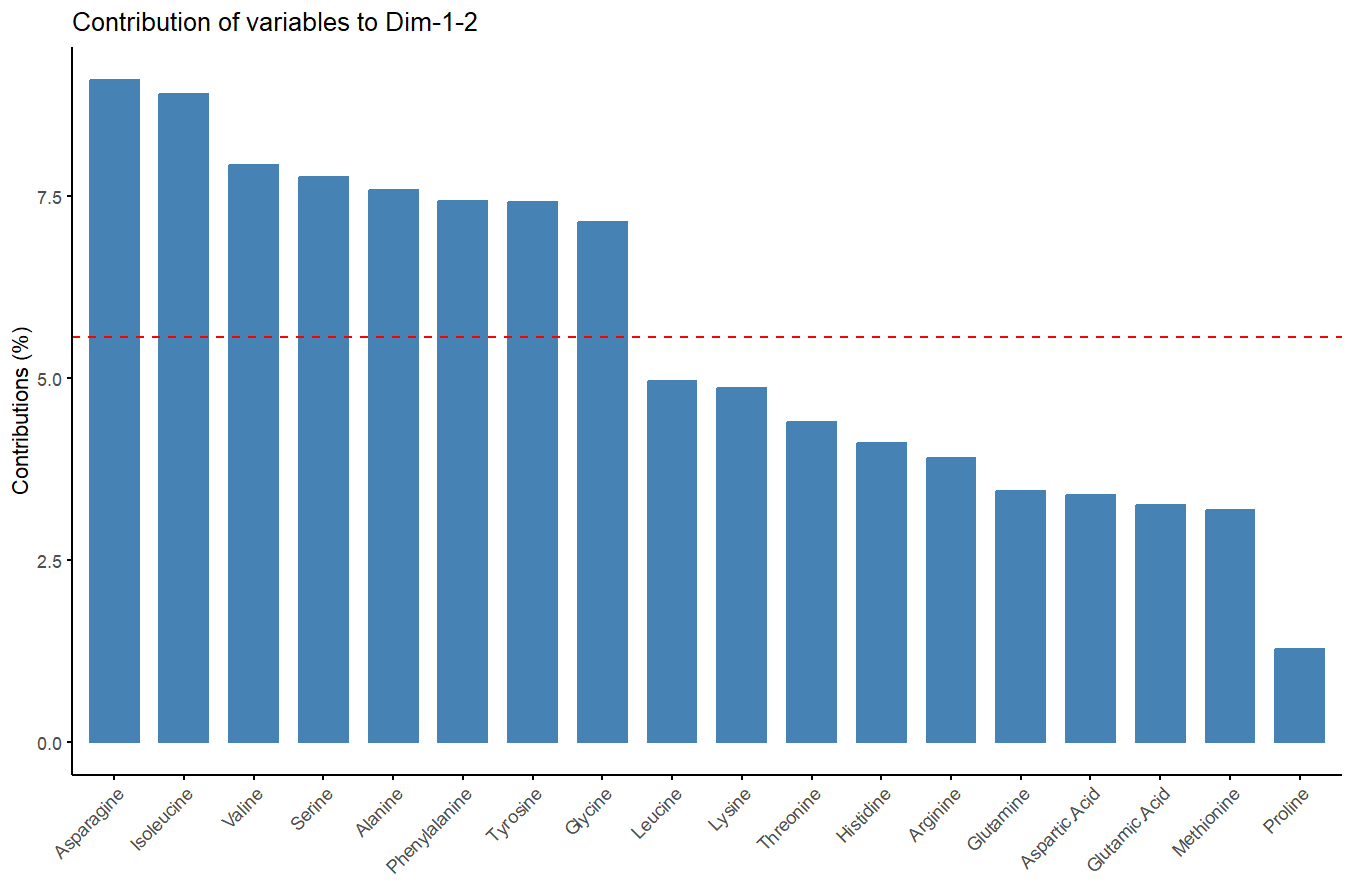


**Supplementary Figure 2:** **Bar plot of** **contributions (%) of free amino acids to dimensions 1 and 2. The red dashed (cutoff) line on the graph indicates the expected average contribution.**
