# Supplementary material for: Genetic architecture of tuber-bound free amino acids in potato and effect of growing environment on the amino acid content
Source: Sci Rep. 2023 Aug 25;13:13940. doi: 10.1038/s41598-023-40880-5 (PMC10457394; doi:10.1038/s41598-023-40880-5)
Supplement: Supplementary file 3 — Supplementary Figure 3. [file 41598_2023_40880_MOESM3_ESM.docx]

**Supplementary Figures**


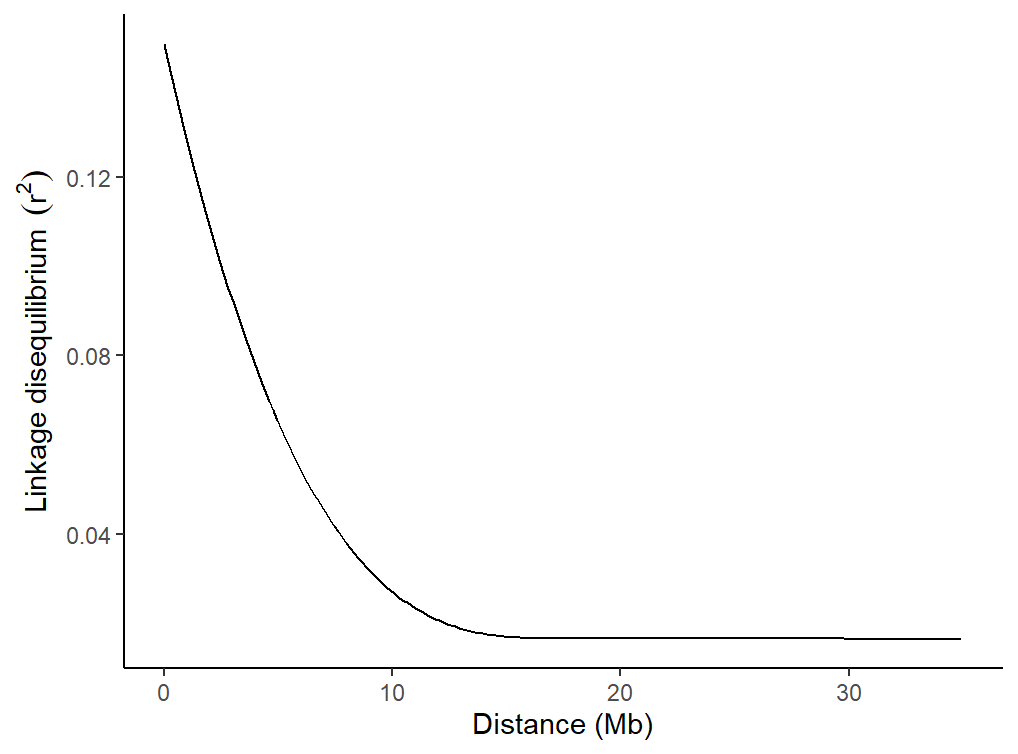


**Supplementary Figure 3:** Genome-wide linkage disequilibrium (LD) decay (in Mb) in 214 potato clones using LD. plot function in GWASpoly. A monotone decreasing, convex spline was fit using the R package scam.
