# Supplementary material for: Genetic architecture of tuber-bound free amino acids in potato and effect of growing environment on the amino acid content
Source: Sci Rep. 2023 Aug 25;13:13940. doi: 10.1038/s41598-023-40880-5 (PMC10457394; doi:10.1038/s41598-023-40880-5)
Supplement: Supplementary file 4 — Supplementary Figure 4. [file 41598_2023_40880_MOESM4_ESM.docx]

**Supplementary Figures**

A) Histidine


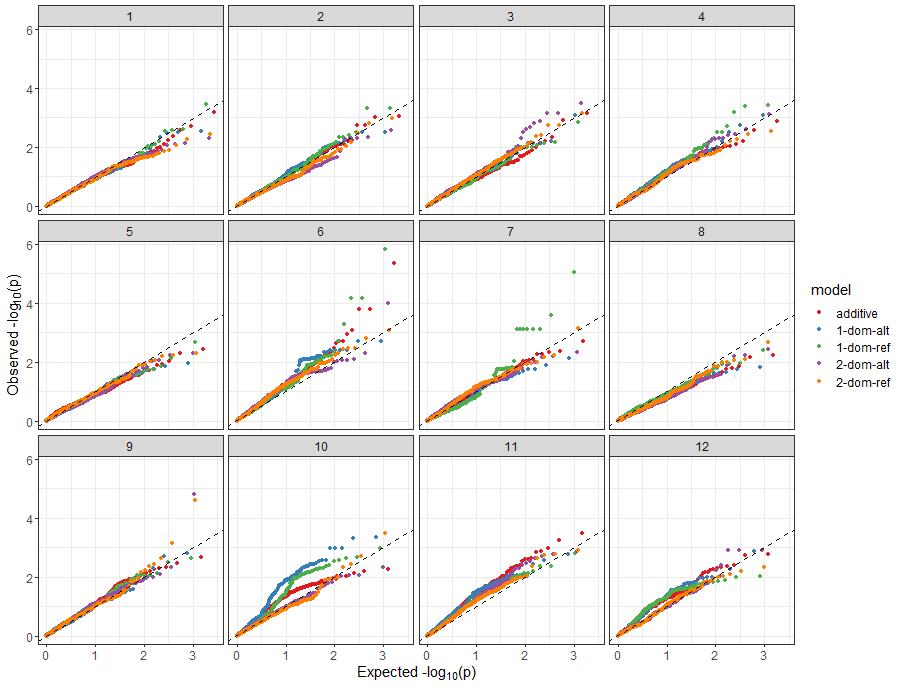


B) Arginine


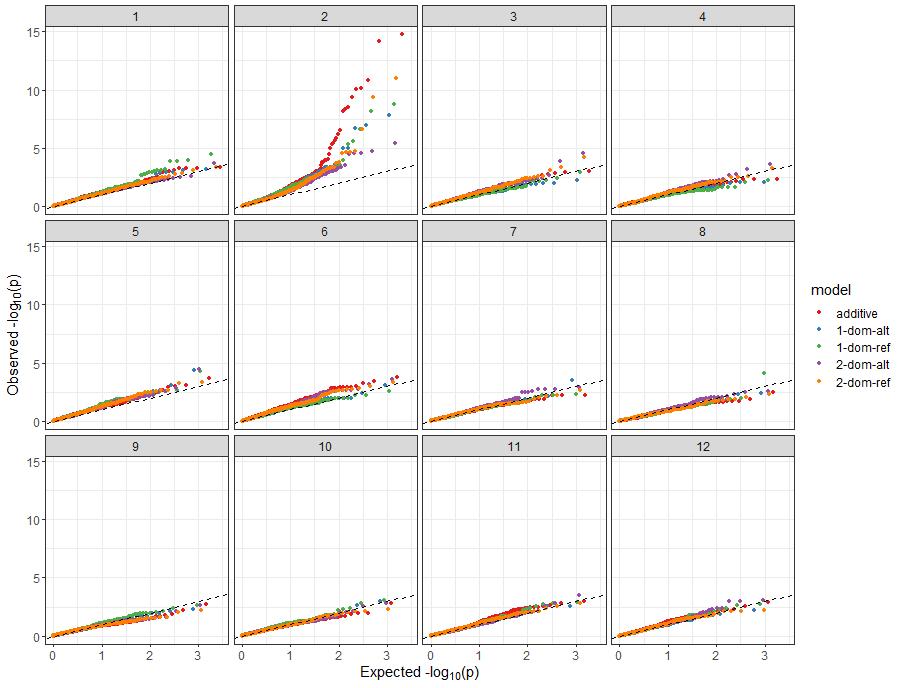


C) Asparagine


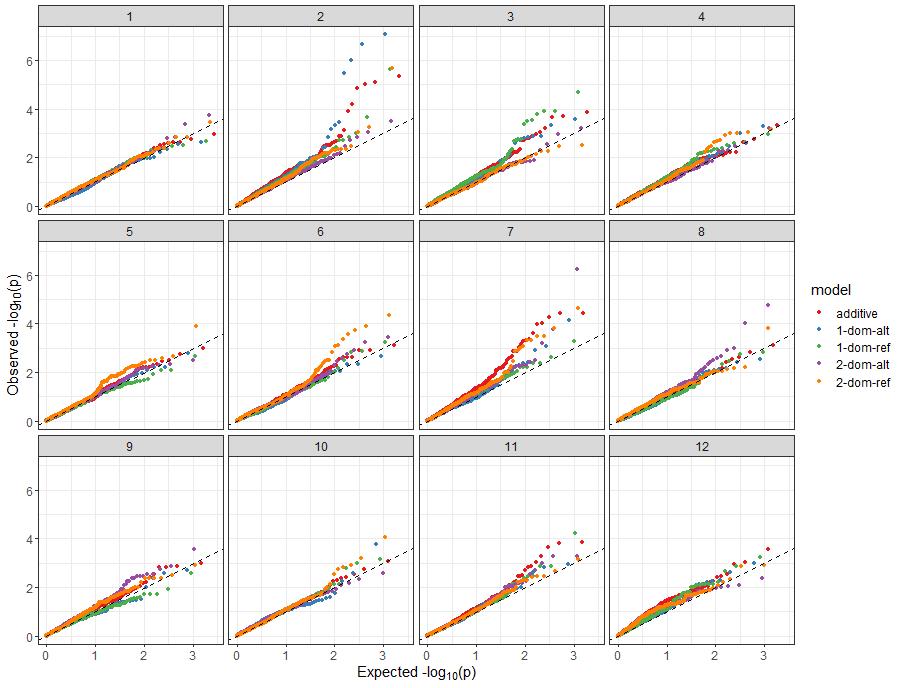


D) Glutamine


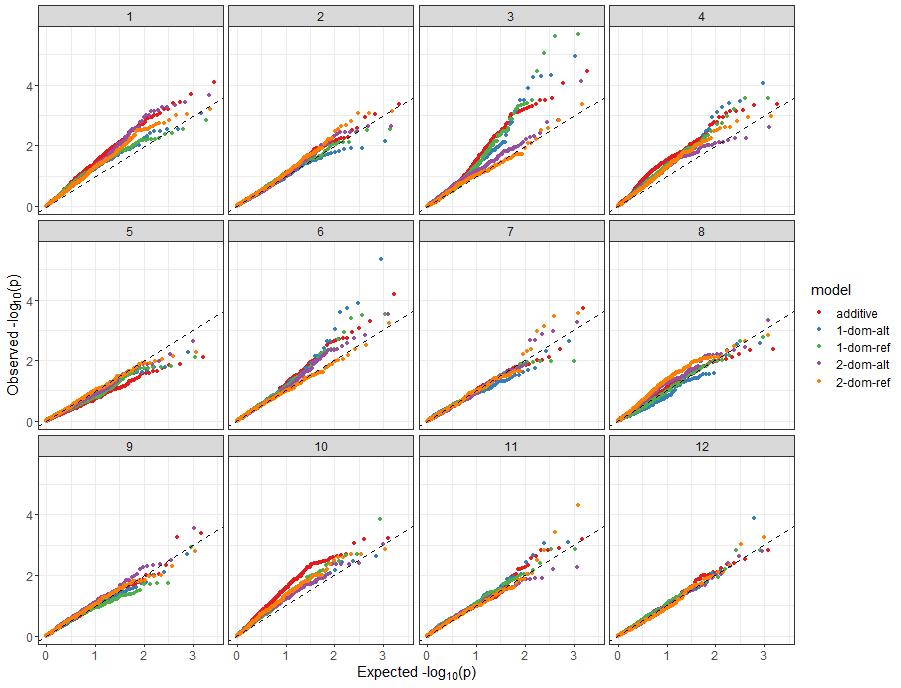


E) Serine


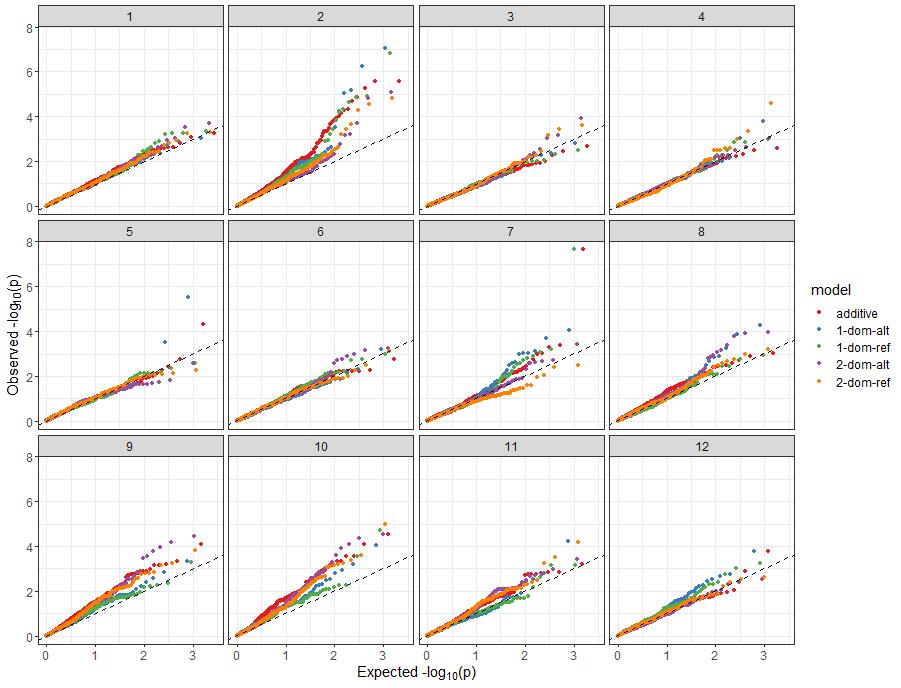


F) Aspartic Acid


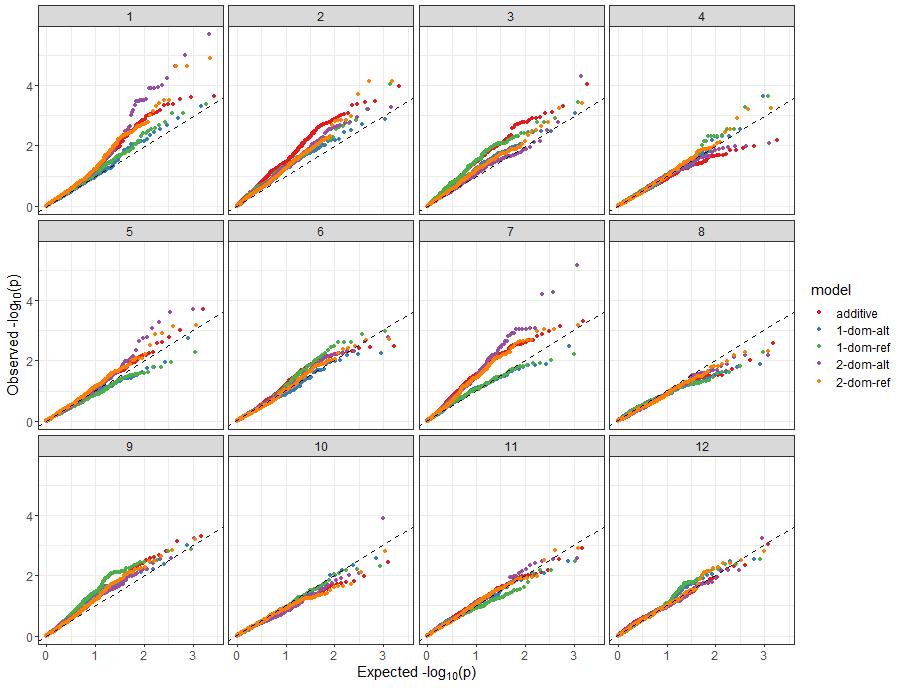


G) Threonine


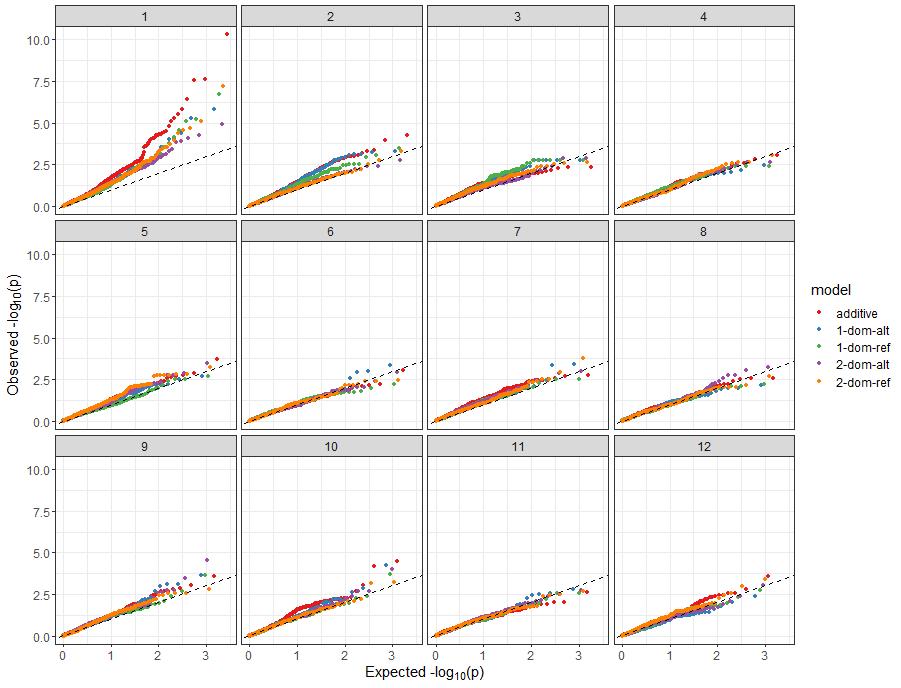


H) Glycine


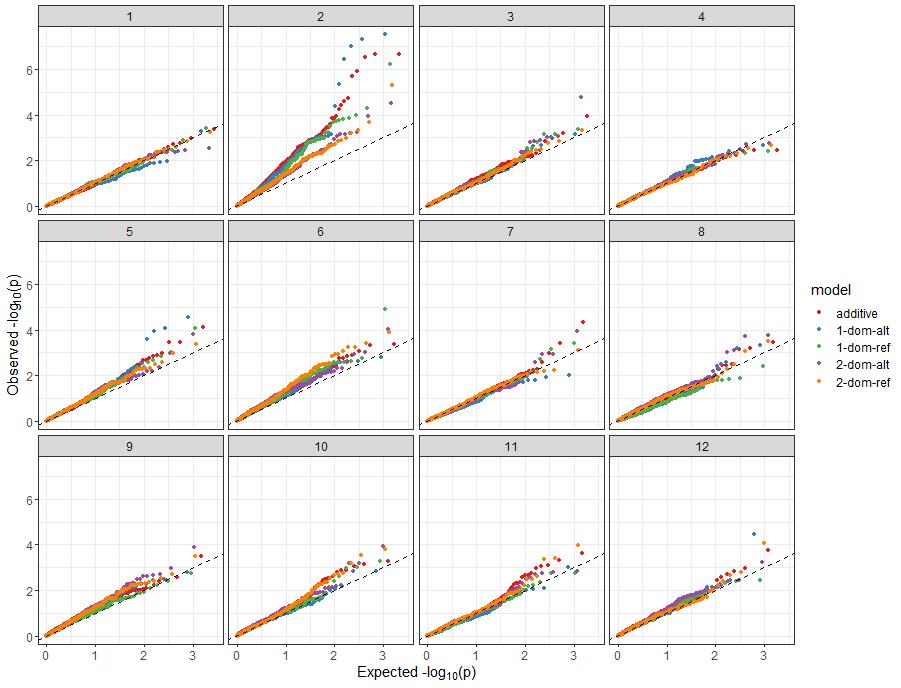


I) Alanine


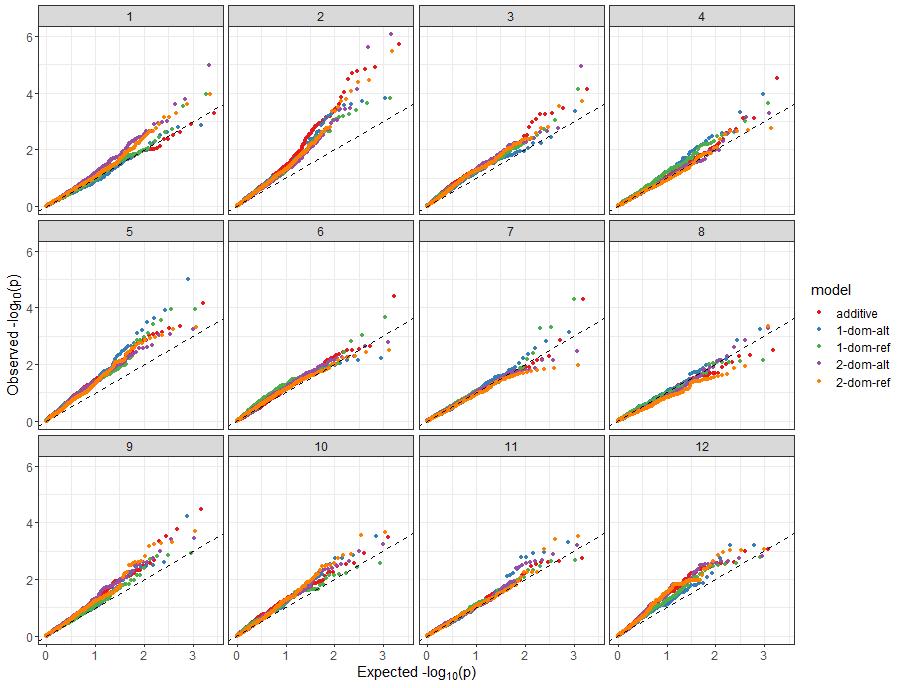


J) Valine


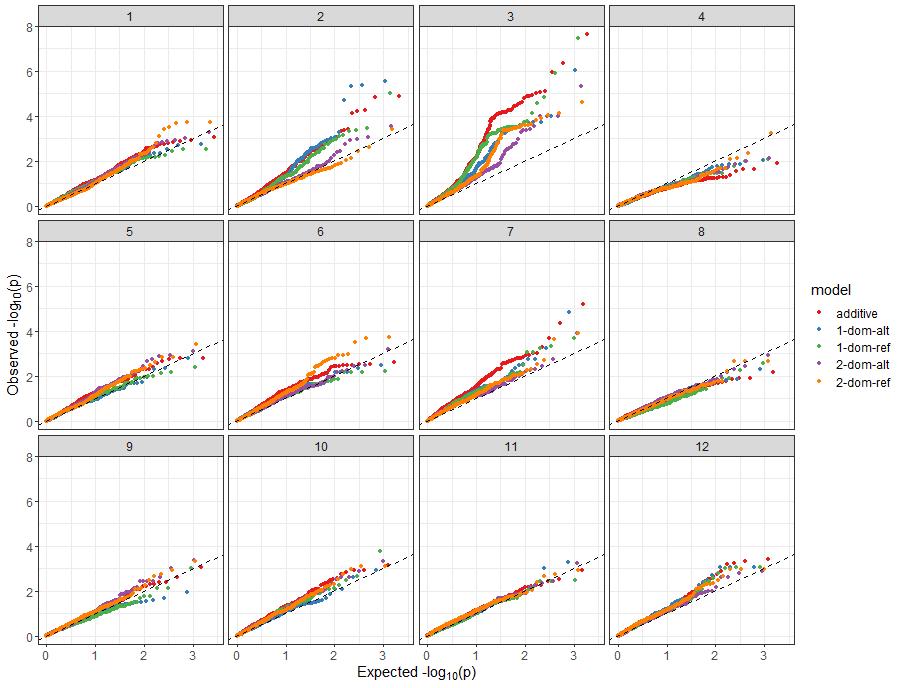


K) Isoleucine


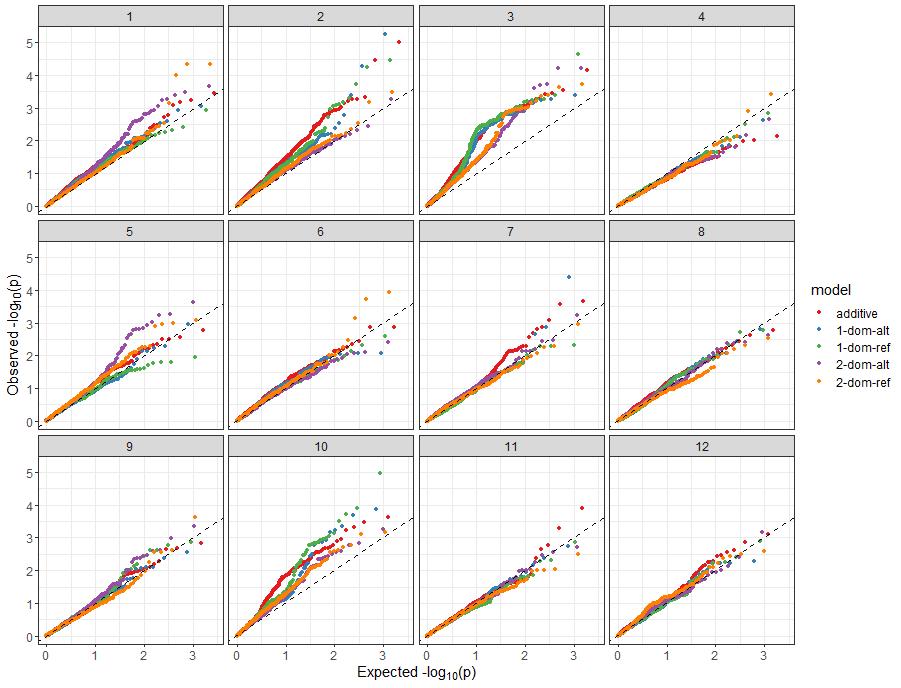


L) Leucine


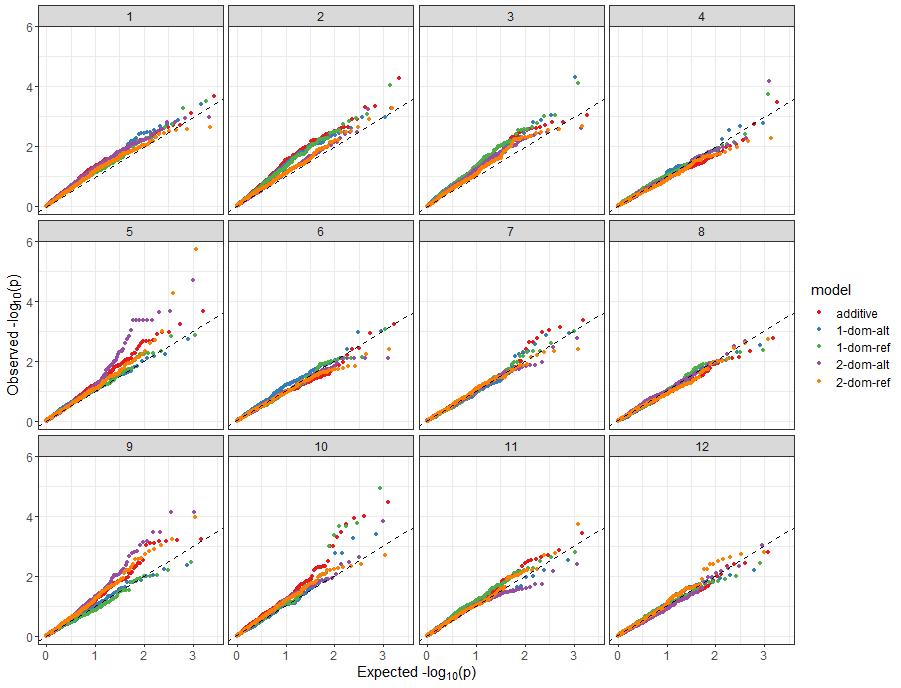


M) Phenylalanine


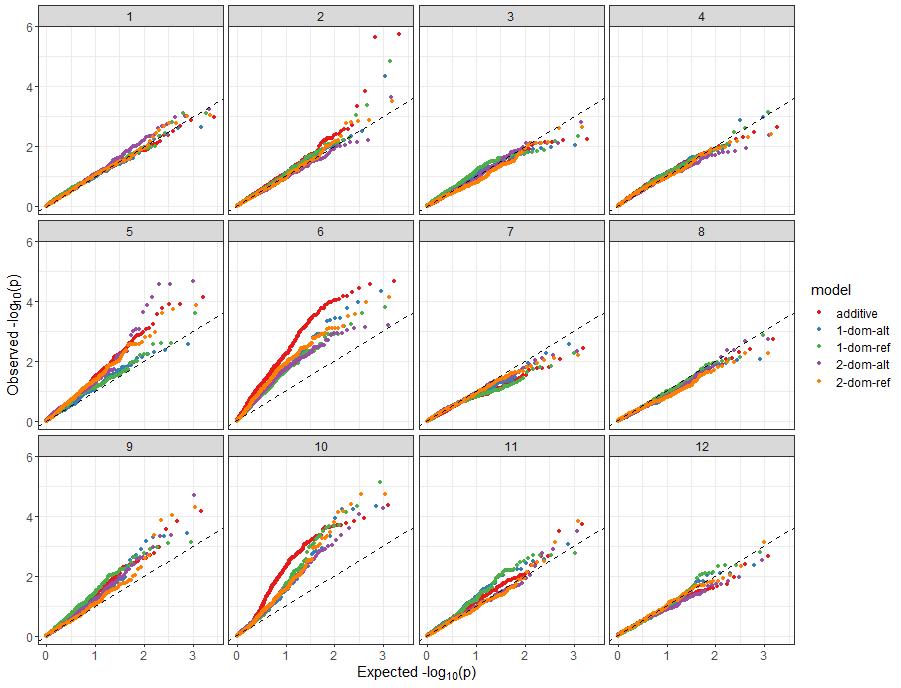


**Supplementary Figure 4:** Q–Q plots of observed versus expected -log10 (P values) for Histidine (A), Arginine (B), Asparagine (C), Glutamine (D), Serine (E), Aspartic Acid (F), Threonine (G), Glycine (H), Alanine (I), Valine (J), Isoleucine (K), Leucine (L), Phenylalanine (M) using the additive and dominant model in three combined environments. The Bonferroni threshold is at 5.31 for the additive, 5.03 for 1-dom-alt, 5.13 for the 1-dom-ref, 5.18 for 2-dom-alt and 5.21 for 2-dom-ref model. The dashed line represents the expected distribution of GWAS scores under the null hypothesis (H_0_).
